# Liver Effects of Clinical Drugs Differentiated in Human Liver Slices

**DOI:** 10.3390/ijms18030574

**Published:** 2017-03-07

**Authors:** Alison E. M. Vickers, Anatoly V. Ulyanov, Robyn L. Fisher

**Affiliations:** 1 Human Translational Models LLC, P.O. Box 4593, Irvine, CA 92612, USA; 2 Inova Translational Medicine Institute, Inova Hospital, Fairfax, VA 22031, USA; 3 Vitron, Inc., Tucson, AZ 85747, USA

**Keywords:** human liver slices, drug injury

## Abstract

Drugs with clinical adverse effects are compared in an ex vivo 3-dimensional multi-cellular human liver slice model. Functional markers of oxidative stress and mitochondrial function, glutathione GSH and ATP levels, were affected by acetaminophen (APAP, 1 mM), diclofenac (DCF, 1 mM) and etomoxir (ETM, 100 μM). Drugs targeting mitochondria more than GSH were dantrolene (DTL, 10 μM) and cyclosporin A (CSA, 10 μM), while GSH was affected more than ATP by methimazole (MMI, 500 μM), terbinafine (TBF, 100 μM), and carbamazepine (CBZ 100 μM). Oxidative stress genes were affected by TBF (18%), CBZ, APAP, and ETM (12%–11%), and mitochondrial genes were altered by CBZ, APAP, MMI, and ETM (8%–6%). Apoptosis genes were affected by DCF (14%), while apoptosis plus necrosis were altered by APAP and ETM (15%). Activation of oxidative stress, mitochondrial energy, heat shock, ER stress, apoptosis, necrosis, DNA damage, immune and inflammation genes ranked CSA (75%), ETM (66%), DCF, TBF, MMI (61%–60%), APAP, CBZ (57%–56%), and DTL (48%). Gene changes in fatty acid metabolism, cholestasis, immune and inflammation were affected by DTL (51%), CBZ and ETM (44%–43%), APAP and DCF (40%–38%), MMI, TBF and CSA (37%–35%). This model advances multiple dosing in a human ex vivo model, plus functional markers and gene profile markers of drug induced human liver side-effects.

## 1. Introduction

Drug induced liver injury often involves various cell types and organelles, and represents the culmination of oxidative stress, endoplasmic reticulum (ER) stress, mitochondrial dysfunction, apoptosis, necrosis and inflammation [1,2,3,4]. A relevant model to investigate drug induced liver injury ex vivo and human response is human liver slices, a 3-dimensional multi-cellular organotypic model in which tissue architecture and function, cell-cell and cell-matrix interactions, including the release of cell mediators, is maintained to mimic in vivo function. Drug concentrations that pose a risk for adverse events in relation to animal study findings can be defined, as well as pathways linked to drug induced injury [5,6,7,8]. These studies strengthen the focus of clinical predictors of adverse events and the identification of safety biomarkers. Serum biomarkers used in concert with serum alanine aminotransaminase values (ALT1), such as α- or pi-glutathione *S*-transferases, or mitochondrial biomarkers like alanine aminotransferase 2 ALT2 and glutamate dehydrogenase (GLDH) or microRNAs and others will provide insight about the type of liver injury [9,10,11,12,13]. Furthermore, these studies provide a means to select the drug candidates with the best safety profile for development.

The metabolic conversion of drugs is an important component of hepatotoxicity and human liver slices generate the full spectrum of drug metabolites as detected in vivo [14,15,16,17,18]. The hepatocytes are the primary site of the drug metabolizing enzymes, however endothelial cells are metabolically active and Kupffer cells possess myeloperoxidase, which can metabolize drugs [19]. Additionally, upon activation endothelial and particularly Kupffer cells contribute to an inflammatory response, releasing either pro-inflammatory cytokines to activate death and survival pathways, or triggering a suppression of inflammation and the release of anti-inflammatory cytokines to inhibit wound repair [20,21]. Hepatic stellate cells, which surround the endothelium, are the major mediators of matrix formation in response to injury [22]. In the presence of drug-induced injury, the tissue response may include aspects of inflammation, repair and regeneration with the outcome determined by the extent of injury and number of hits [23,24]. Human liver slices cultured for several days exhibit the activation of endothelial cells, Kupffer cells, stellate cells and repair pathways [25,26]. Advancements in the culturing of human liver slices to extend the viability of human liver slices to several days has contributed to the evaluation of time-dependent changes, likely due to the formation of minor metabolites and the manifestations of the consequences [8,26,27].

In this study, the ex vivo human liver slice model was used to characterize the initial effects of drugs associated with liver adverse effects clinically which encompass diverse mechanisms contributing to liver dysfunction and injury. All drugs were dosed daily and compared side-by-side within each human liver, either at clinical serum/plasma concentrations linked with altered liver function or at concentrations known to alter human liver slice function [8,28]. Some of the drugs selected were linked with oxidative stress which could impact liver glutathione status and/or mitochondrial function and ATP status, including acetaminophen (APAP, 1 mM), diclofenac (DCF, 1 mM), and methimazole (MMI, 500 μM). Drugs known to interact directly with mitochondria, and hence, could affect mitochondrial function, as well as cause general cell stress included, cyclosporin A (CSA, 10 μM), dantrolene (DTL, 10 μM), and etomoxir (ETM, 100 μM). Drugs considered to affect liver function via oxidative stress and linked with hypersensitivity reactions included terbinafine (TBF, 100 μM) and carbamazepine (CBZ, 100 μM) [29,30,31]. Overall, in this study, time-dependent differences in the functional markers of oxidative stress, liver slice ATP and GSH levels (24–72 h), supported the use of daily dosing to reveal persistent and statistically significant differences. Gene expression changes indicative of organelles (mitochondrial energy, ER stress) or pathways linked with organ dysfunction (oxidative stress, fatty acid metabolism, apoptosis) and toxicity (DNA damage and repair, cholestasis, necrosis, immune and inflammation) further characterized human response to the drugs, and the mechanisms underlying liver side effects. The spectrum of changes induced by each drug and across drugs can serve as a means to evaluate the safety and dose limitations of unknown compounds for potential side effects, as well as to identify biomarkers of organ injury for more accurate forecasts of drug-induced changes clinically.

## 2. Results

**Figure 1:**
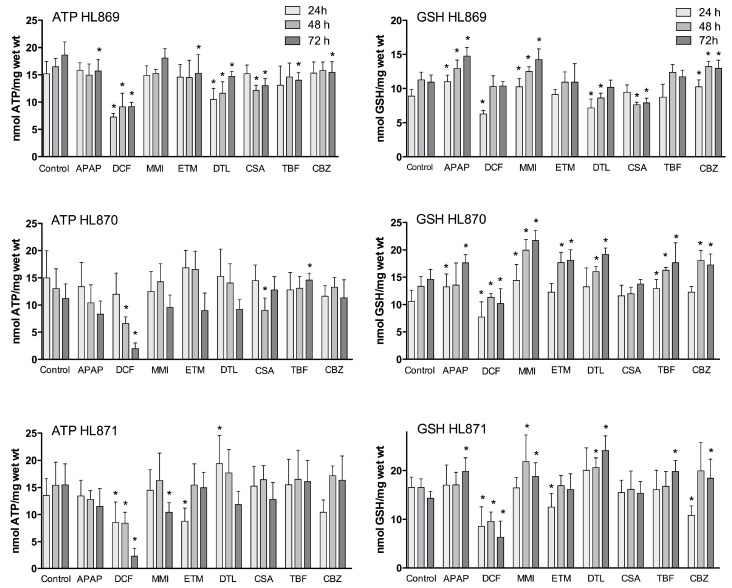
Time course of ATP and GSH levels (24–72 h) in human liver slices exposed to daily dosing of APAP (1 mM), DCF (1 mM), MMI (500 μM), TBF (100 μM), CBZ (100 μM), ETM (100 μM), DTL (10 μM), CSA (10 μM). Values were determined in 10 control slices or 6 treated slices/time-point/liver. Statistical significance, *p* < 0.05, is labeled (*) and related to the time-matched control value.

Functional markers of oxidative stress include liver slice ATP and GSH levels. The mitochondria have an essential role in energy metabolism and as a regulator of cell death. GSH is a major liver anti-oxidant and if GSH redox status is compromised reactive metabolites will bind to cell proteins to affect cell function. Utilization of a complete culture medium contributed to the liver slices ability to synthesize ATP and GSH throughout the culture period (Figure 1). In the control human liver slices, ATP mean values across the three livers were comparable (13–16 nmols/mg wet weight) and remained consistent over the time course, varying 10% for HL869, 14.5% for HL870, and 7% for HL871. GSH mean control values were also comparable across the three livers, ranging from 10–15 nmols/mg wet weight. The variation within each liver across the 72 h incubation period, 12% for HL869, 16% for HL870, and 8% for HL871, paralleled the variation measured for ATP values. The quality of each human liver was initially considered to be very good as determined by high liver slice K^+^ levels at 1 and 4 h and was verified by additional measurements at 24, 48, and 72 h. Liver slice K^+^ levels were sustained for 72 h, varying 6.5% for HL869, 4.3% for HL870, and 6.4% for HL870 (Table 1). The ATP and GSH levels of the individual human liver slice experiments revealed inter-individual differences to drug response and some similar patterns (Figure 1). For example, after three doses (72 h) some drugs decreased ATP levels in each liver (APAP, DCF, and DTL), while some drugs reduced ATP levels in two livers (MMI, ETM, CSA) or in one liver (TBF, CBZ). GSH levels after three doses (72 h) were increased in each liver by APAP, MMI, TBF and CBZ, while some drugs increased levels in two livers, ETM and DTL. GSH levels were decreased in two livers by DCF and CSA. To provide an overview, mean ATP and GSH values based on percent of change from time-matched control values are compared across the three livers in Table 2.

### 2.1. Drugs Affecting ATP

**Figure 2:**
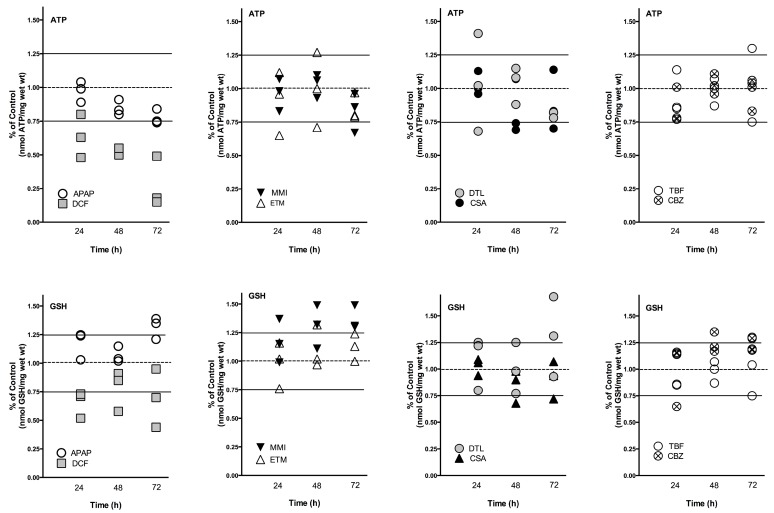
Human liver slice ATP and GSH mean levels are represented as percent of control (24, 48, 72 h) for the 3 individual livers to compare drug response across the livers.

A time dependent decrease of ATP levels was evident with 1 mM APAP and 1 mM DCF compared to time-matched control slices (Figure 2). In the presence of 1 mM APAP, mean liver slice ATP levels decreased significantly at 48 h by about 20% in two livers, and at 72 h by 16%–25% in the three livers. The dose of DCF (1 mM) decreased liver ATP levels dramatically in the three livers, up to 50% at 24 and 48 h and 50%–85% by 72 h.

Several compounds caused fluctuations in ATP levels compared to time-matched controls. MMI (500 μM) increased ATP levels by 10% above control levels at 24 and 48 h in two of three livers, then decreased ATP levels significantly by 14%–33% at 72 h. ETM (100 μM) caused fluctuations in ATP production in all three livers. At 24 h ATP levels were significantly decreased by 35% in one liver, which then readjusted to control values for the remainder of the culture period. In two livers at 48 h, one exhibited increased ATP production (27%) while another liver exhibited a 30% decrease. At 72 h, ATP levels in two of three livers were decreased by 20%.

DTL (10 μM) caused the greatest fluctuation in ATP levels at 24 h, increasing by 41% or decreasing by 32%. At 48 h the liver stabilized to within 12%–15% of control values. At 72 h all three human livers exhibited decreased ATP production, with a significant mean decrease of 19%. CSA (10 μM) stimulated ATP production in one liver at 24 h (13%), then caused decreases in ATP levels in two of three livers at 48 h (26%–31%) and 72 h (17%–30%) and an increase in one liver at 72 h (14%).

TBF (100 μM) initially increased or decreased liver ATP levels by 15% at 24 h, which stabilized to control levels at 48 h, then decreased significantly in one liver by 25% and increased significantly in another liver by 30% at 72 h. CBZ (100 μM) initially decreased ATP levels up to 23% in two livers at 24 h, which then stabilized around control values at 48 h, and one liver exhibited a significant decrease of ATP levels by 17% at 72 h.

### 2.2. Drugs Affecting GSH

APAP (1 mM) exposure to human liver slices increased GSH levels significantly in two livers at 24 h (up to 25%) and in the three livers significantly at 72 h (21%–39%) compared to time matched controls (Figure 2). DCF (1 mM) significantly reduced human liver slice GSH levels at 24 h in all three livers, 50%–73%. Recovery of GSH levels was apparent in one liver, with 91% of control values at 48 h, and 95% at 72 h. Another liver exhibited partial recovery at 48 h, 85% of control values, then GSH levels declined significantly to 70% at 72 h. A third liver had significantly reduced GSH levels (about 50%) at all time points.

MMI (500 μM) exposure increased GSH levels significantly at 24 h (15%–37%) in two of three livers. GSH levels remained elevated and were significantly increased in the three livers at 48 h (11%–49%) and at 72 h (30%–49%). ETM (100 μM) increased GSH levels significantly in one liver at 48 h (32%) and 72 h (24%). Another liver decreased ATP levels significantly initially (24%) at 24 h, then exhibited elevated levels at 72 h (13%).

DTL (10 μM) increased GSH levels in two of three livers, up to 25% at 48 h and up to 68% at 72 h. However, in one liver DTL decreased GSH levels significantly 20%–23% at 24 and 48 h, which rebounded to control levels by 72 h. CSA (10 μM) significantly decreased GSH levels in one of three livers, about 30% at 48 and 72 h; while the other livers had values that were equal to control slices.

TBF (100 μM) increased GSH levels, reaching significance, 20% 24–72 h in one liver, and 39% at 72 h in a second liver. CBZ (100 μM) overall increased GSH levels significantly, up to 16% at 24 h and 20%–30% at 48 and 72 h in two livers. There was an initial decrease of GSH levels at 24 h (35%) in one liver, which then increased to 30% above control values at 72 h.

### 2.3. Fluctuations in Drug Response

Fluctuations in ATP and GSH levels occurred across the time-course to the drugs in each liver, which is indicative of a system adjusting to drug exposure. Additionally, inter-individual variation in response to drug exposure was apparent across the livers (Figure 2, Table 2). For example, ETM and DTL exposure caused an initial decrease in ATP levels at 24 h which was followed by an increase at 48 h, which then decreased by 72 h. A considerable spread in response across the three livers was also measured at 24 h with DTL (0.68–1.41 percent of control), and at 48 h with ETM exposure (0.7–1.27 percent of control). The doses of APAP, DCF, MMI, CSA, TBF, and CBZ caused less fluctuation in response across time-points; however inter-individual variation was apparent.

Human liver slice GSH levels were increased substantially by APAP (up to 39%), MMI (up to 49%), and DTL (up to 68%), and substantially decreased by DCF (50%) exposure. Fluctuations in GSH levels were evident across the time course. An initial decrease of GSH levels (24 h) was followed by an increase at 48 h compared to time-matched controls for DCF, and in one liver for ETM and CBZ. In contrast, APAP induced an initial increase at 24 h in GSH levels followed by a decrease at 48 h, and then an increase at 72 h. MMI caused a general increase in GSH levels across the time course, while CBZ increased GSH levels at 48 and 72 h. Inter-individual response was most evident with DCF and DTL exposure, particularly at 72 h. Drug exposure exhibiting a tighter response included CBZ, CSA, ETM and APAP.

### 2.4. Gene Expression

**Figure 3:**
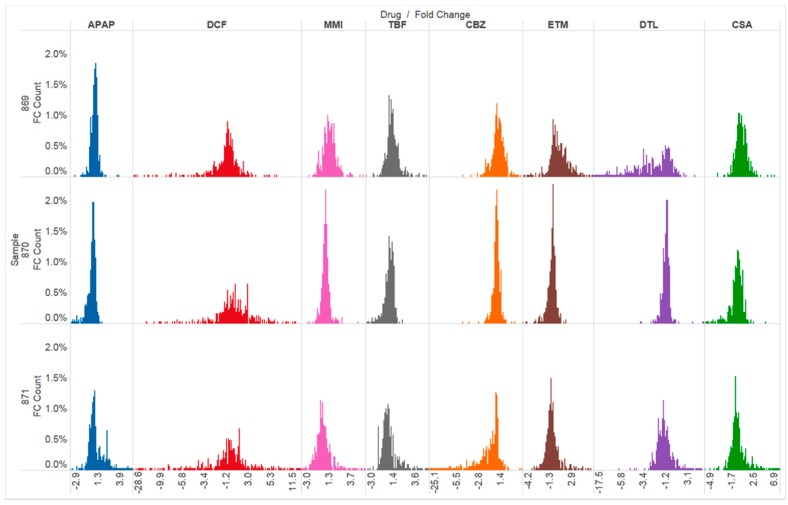
Histograms of fold change and distribution of the 370 genes queried with the human specific Molecular Toxicology Pathway Finder PCR array. Each column corresponds to a drug and each row corresponds to the individual human livers (869, 870, 871). The global response to the drugs has the same distribution across the drugs, except for DCF and DTL in human liver 869 in which a greater number of genes were altered.

**Figure 4:**
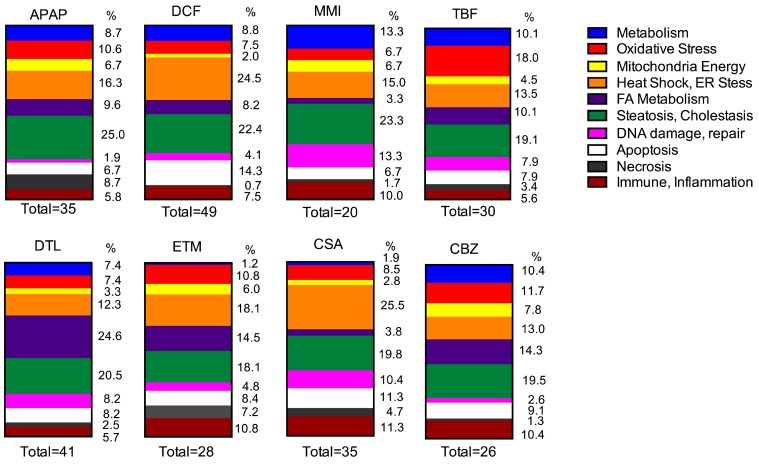
The gene categories for each drug are arranged to compare drug response of the 8 drugs used in this study. The percentage of genes represented by each category is based on the number of significant total gene changes for each drug, which is listed at the bottom of each bar graph.

Genes linked to organ injury were interrogated following two doses of each drug and compared to time-matched control liver slice RNA with the molecular toxicology pathway finder human PCR array (PANZ-3401, 370 genes). Exposure of the eight drugs to three independent human livers reveals differences across the drugs in effects as well as differences in human response across the livers. Changes in gene expression reflect a perturbation of organelles and tissue stresses. A comparison of the gene categories altered by each drug is shown in Figure 3 and Figure 4. Furthermore, an overview of the number of genes altered as well as the list of the genes significantly altered by each drug is shown in Table 3 and Table 4.

### 2.5. Metabolism

Several drugs altered the expression of the drug metabolism genes including enzymes that play a role in their own metabolism. DCF, TBF, CBZ, APAP, MMI and DTL affected several genes, while ETM and CSA had minimal effects on the metabolism genes. *CYP3A4* was up-regulated by TBF (1.7–10.5-fold) and CBZ (1.9–3.0-fold) in two different livers, and in one liver by APAP (8.4-fold), and CSA (4.7-fold). A down-regulation of *CYP3A4* occurred with DCF (−11-fold), ETM (−8.7-fold) and DTL (−5.5-fold) exposure. *CYP2C19* gene expression, was down-regulated by DCF (−2.1 to −28.6-fold) in all three livers; while up-regulated by CBZ (3-fold) in two livers. DTL altered *CYP2C19* expression differently in two livers. MMI up-regulated *CYP2C19* and *CYP2B6* in the same liver and down-regulated these genes in a second liver. APAP up-regulated *CYP2B6* (1.7–2.4-fold) in two livers, and down-regulated *CYP2E1* in the one liver. DCF, additionally, down-regulated *CYP2B6* (−22-fold) gene expression in one liver, and caused down-regulation of *FMO4* (up to −6.4-fold) in the three livers and *FMO5* (−7.2-fold) in one liver. DTL down-regulated *FMO3* (up to −6.3-fold) in two livers. CSA also caused down-regulation of *CYP2C9* in one liver.

The cytochrome P450 reductase (*POR*) gene, which encodes for the enzyme that transfers electrons from NADPH to the various cytochrome P450s, was up-regulated by DCF (2.5 to 3.5-fold) in all three livers, and by TBF (2.8-fold) in one liver. Several drugs caused an up-regulation of *CYP1A1* and *CYP1A2* gene expression. TBF up-regulated both genes (2.2 to 4.5-fold) in all three livers; while MMI (2.6 to 7.8-fold) and DTL (7.4 to 9.6-fold) caused up-regulation of these genes in two livers. APAP and CBZ up-regulated *CYP1A1* in one liver and down-regulated it in another liver. DCF exposure down-regulated *CYP1A2* (−70-fold) in one liver.

### 2.6. Oxidative Stress

Oxidative stress is often an initial consequence of reactive intermediate formation, and several of the drugs in this study undergo metabolic conversion; hence, many oxidative stress genes were affected. DCF altered 3 of 5 genes (7.5%) in all three livers. The same gene changes in two livers occurred with TBF, 6 of 10 genes (18%), CBZ, 3 of 6 genes (11.7%), DTL, 2 of 6 genes (7.4%), CSA, 2 of 7 genes (8.5%), ETM, 1 of 8 genes (10.8%), and MMI, 1 of 3 genes (6.7%). Gene changes in one liver occurred with APAP (11 genes, 10.6%).

Evidence for effects on glutathione regulation was apparent by altered expression of glutathione transferase (*GSTA3*) for APAP (−3.1-fold), DCF (−34.4-fold), TBF (−2.3 to 2.0-fold), CBZ (3.4-fold), ETM (−2.0 to −4.4-fold). In particular, the mu class of GST enzymes (*GSTM4*) was altered by DCF (−3.1 to −7.5-fold) in all three livers. MMI and CSA had no effect on either *GSTA3* or *GSTM4*. Various gene isoforms of glutathione peroxidase, the intracellular *GPX1* and *GPX2* and extracellular *GPX3* are responsible for the majority of the glutathione-dependent hydrogen peroxide-reducing activity, were altered particularly by TBF (1.8 to 3.1-fold) and CBZ (1.5 to 2.6-fold), followed by DTL (−2.0-fold), CSA (−3.4-fold), APAP (−1.6 to 1.5-fold), DCF (−15.9-fold), and ETM, while MMI had no effect. Genes indicative of reactive intermediate formation, microsomal epoxide hydrolase 1 (*EPHX1*) which converts epoxides to diols was altered by DCF (−2.2 to −8.9-fold) in all three livers, followed by CBZ (1.6 to 1.8-fold), TBF (2.5-fold) and APAP (1.6-fold). Reactive oxygen formation is indicative of dual oxidase (*DUOX1-2*) changes, which was altered by TBF (1.8–2.3-fold), CBZ (−2.1 to 4-fold), CSA (2.5 to 4.3-fold) and APAP (−2.3 to −3.1-fold). Up-regulation of the *NQO1* gene which encodes for NAD(P)H dehydrogenase (quinone 1), and is involved in the reduction of quinones to hydroquinones, was evident with MMI (1.9 to 2.9-fold) and TBF (2.6-fold). Additionally, MMI up-regulated the *PRDX1* gene (3.7-fold), which encodes for a member of the peroxiredoxin family of antioxidant enzymes to reduce hydrogen peroxide and alkyl hydroperoxides. The gene *PPP1R15B* encodes for a phosphatase, that regulates a translation factor, was affected by DCF (2.0–26.4-fold) in all three livers, followed by TBF (−2.0 to 9.3-fold). The liver (HL871) that exhibited the greatest up-regulation for DCF (26.4-fold) was affected by several drugs, yet to a lesser extent, APAP 7.3-fold, MMI 8.2-fold, ETM 7.5-fold, DTL 8.2-fold and CSA 8.6-fold.

### 2.7. Mitochondrial Energy

An important category that gene expression can provide insight into is the effects of drugs on mitochondrial pathways. This category includes genes encoding for enzymes involved with the TCA cycle and mitochondrial energy. DCF affected only 1 gene in all three livers (2%). APAP altered 2 genes of 5 in two livers (6.7%), TBF 1 of 3 genes (4.5%) and 1 of 4 genes ETM (6%) in two livers, followed by changes in one liver by CBZ (6 genes, 7.8%), MMI (4 genes, 6.7%), DTL (4 genes, 3.3%) and CSA (3 genes, 2.8%).

The aconitase genes, *ACO1* and *ACO2*, encode for proteins catalyzing steps in the TCA cycle. *ACO1* aids to control iron levels inside cells and was affected in the same liver by MMI (−1.7-fold), TBF (−2.6-fold), CBZ (−3.7-fold), ETM (−2.5-fold), DTL (−2.1-fold), and CSA (−2.9-fold), while *ACO2* was altered in a different liver by APAP (−1.5-fold). Genes which encode for enzymes that utilize NAD(+) and NADP(+) as electron acceptors the *IDH 1-3* (isocitrate dehydrogenases) were altered. MMI (−1.7-fold) down-regulated *IDH1*, while APAP (−1.7-fold) and CBZ (−1.7-fold) down-regulated *IDH2*. APAP (1.3-fold) up-regulated *IDH3B*, while ETM (−1.8-fold) down-regulated *IDH3B*. The *MDH1* gene encodes for a cytosolic enzyme malate dehydrogenase, which utilizes the NAD/NADH cofactor system in the citric acid cycle. The *MDH1* gene was altered by APAP (−1.5 to 3.1-fold), MMI (3.1-fold), TBF (−1.9 to 3.6-fold), CBZ (−2.9-fold), ETM (−4.2 to 3.6-fold) and CSA (−1.9-fold). Furthermore, the *SDHC* gene, succinate dehydrogenase complex subunit C also known as mitochondrial complex II, encodes for a key enzyme complex of the TCA cycle and aerobic respiratory chains of the mitochondria, was down-regulated by CBZ (−5.7-fold) and DTL (−1.7-fold). *SUCLA2* and *SUCLG1* are genes that encode for mitochondrial matrix succinyl-CoA ligases which are accompanied by the substrate-level phosphorylation of ADP to ATP or GDP to GTP. Exposure to CBZ (−10.3-fold) down-regulated *SUCLA2*, and APAP (−1.4 to 3.3-fold) altered *SUCLG1* gene expression levels.

The *ACYL* gene, which encodes for the enzyme ATP citrate lyase, is responsible for the synthesis of cytosolic acetyl-CoA was up-regulated by CSA (2.3-fold). The adenosine kinase (*ADK*) gene, which encodes for an enzyme that catalyzes the transfer of the gamma-phosphate from ATP to adenosine, as well as *COX8A*, which encodes a subunit of the cytochrome c oxidase complex of the mitochondrial respiratory chain were down-regulated by DTL (−5.8-fold, ADK and −6.1-fold, COX8A) in one liver. The mitochondrial uncoupling proteins (UCPs) separate oxidative phosphorylation from ATP synthesis with energy dissipated as heat, and referred to as the mitochondrial proton leak. DCF exposure caused a down-regulation of *UCP2* (−2.4 to −5.1 fold) in all three livers. The *CYC1* gene, cytochrome c1, encodes for a mitochondrial heme protein, which was up-regulated by MMI (1.4-fold) and TBF (1.3-fold) in the same liver. The *DLD* gene, dihydrolipoamide dehydrogenase, encodes for a mitochondrial enzyme that is part of several multi-enzyme complexes involved with energy metabolism. CBZ exposure caused a down-regulation of *DLD* gene expression levels (−27.3-fold).

### 2.8. Fatty Acid Metabolism

Drugs affecting the pathways of fatty acid metabolism have the potential to alter the energy status of the tissue. In this study, the drugs which affected genes in all three livers included: DCF altered 3 of 6 genes (8.2%), APAP altered 1 of 7 genes (9.6%), CBZ altered 1 of 9 genes (14.3%), TBF altered 1 of 5 genes (10.1%), and ETM altered 1 of 8 genes (14.5%), followed by CSA (3.8%) and DTL (24.6%) in which two livers responded, and MMI (3.3%) whereby one liver responded.

Several genes affecting mitochondrial fatty acid oxidation were altered. The *CPT1A* gene encodes for carnitine palmitoyltransferse 1, which is located on the outer mitochondrial membrane, is a rate-limiting step involved in fatty acid metabolism. *CPT1A* was up-regulated in all three livers by ETM (2.3 to 16.1-fold), and up-regulated by TBF (1.5 to 8.1-fold) in two livers, and by APAP (2.1-fold), CBZ (2.1-fold) and CSA (2.5-fold) in the same one liver. The *ACADM* (acyl-coenzyme A dehydrogenase) gene encodes for a mitochondrial enzyme that catalyzes the initial step of medium-chain fatty acid beta-oxidation. *ACADM* gene expression was altered by TBF (−2.1 to 3.4-fold), APAP (−1.9 to 3.2-fold), ETM (4.1-fold), CSA (2.5-fold), and DTL (−5.2-fold). The *ACADVL* (very long-chain specific acyl-CoA dehydrogenase) gene encodes for an inner mitochondrial protein to catalyze the first step of fatty acid beta-oxidation of long chain and very long chain fatty acids, typically C16-acylCoA and longer. An up-regulation of *ACADVL* was triggered by TBF (1.7–2.7-fold), CBZ (3.3-fold), ETM (1.8-fold) and CSA (1.9-fold). The ECHS1 gene, enoly-coA hydratase short chain 1, encodes for a protein that catalyzes the second step of the mitochondrial fatty acid beta-oxidation pathway. DTL down-regulated *ECHS1* (−2.4 to −4.9-fold) in two livers. The *HADHA* gene, the α subunit, and *HADHB*, the beta subunit gene encodes for 3-ketonacyl-CoA thiolase, the trifunctional mitochondrial enzyme that catalyzes the last steps of mitochondrial beta-oxidation of long chain fatty acids. The *HADHA* and *HADHB* genes were up-regulated by TBF (2.1 to 3.2-fold) in one liver and were down-regulated by DTL (−1.5 to −3.1-fold) in two livers. The *ACAA1* gene encodes for acetyl-Coenzyme A acyltransferase 1, which is operative in the beta oxidation system of peroxisomes; whereas the *ACAA2* gene encodes for acetyl-Coenzyme A acyltransferase 2, to catalyze the last step of mitochondrial fatty acid beta oxidation. ETM up-regulated *ACAA1* (1.8 to 4.7-fold) and *ACAA2* (2.4-fold), while DTL down-regulated *ACAA1* (−2.8 to −34-fold) and *ACAA2* (−2.5 to −5.9-fold) gene expression.

Additionally, genes affecting peroxisomal fatty acid oxidation were altered. Three ACOT genes were altered. The *ACOT1* gene, acyl-coenyzme A thioesterase 1, encodes for an enzyme that regulates intracellular levels of CoA esters, Coenzyme A, and free fatty acids to modulate lipid metabolism. The *ACOT8* gene encodes for a peroxisomal thioesterase, which is more involved with the oxidation of fatty acids rather than in their formation. In addition, the *ACOT12* gene encodes for a lipid transfer protein. APAP up-regulated ACOT1 (2.3-fold) and ACOT 8 (1.6-fold); whereas, *ACOT12* was down-regulated by APAP (−1.4-fold), DCF (−3.9-fold) and DTL (−11.2-fold), and up-regulated by CBZ (1.6-fold). The *ACOX2* gene, encodes for the branched-chain acyl-CoA oxidase, which is involved in the degradation of long branched fatty acids and bile acid intermediates in peroxisomes. DCF down-regulated this gene in the three livers (−4.4 to −12.5-fold), while CBZ (1.5-fold) up-regulated the gene in one liver. The *EHHADH* gene, enoyl-CoA hydratase, encodes for a bifunctional enzyme and is one of the four enzymes of the peroxisomal beta-oxidation pathway. The *EHHADH* gene was down-regulated by DTL (−1.8 to −5.4-fold) in two livers, and by CBZ (−14.7-fold) in one liver, and up-regulated by ETM (1.6-fold) in one liver.

The *ACAT2* gene encodes for a cytosolic enzyme involved in lipid metabolism, cytosolic acetyl-CoA acetyltransferase. *ACAT2* gene expression was down-regulated by DCF (−1.9 to −3.5-fold) in the three livers, and by DTL (−2.2 to −4.4-fold) in two livers. The *APOE* gene encodes for the apolipoprotein E protein, produced primarily in the liver, and mediates cholesterol metabolism. APOE is polymorphic, which alters its structure and function, and has physiological consequences. APAP (−1.7 to 3.3-fold) altered *APOE* gene expression in all three livers, while CSA (3.4-fold) altered it in one liver. The *APOF* gene encodes for apolipoprotein F, which is synthesized in the liver and found in plasma to form complexes with lipoproteins involved with the transport of cholesterol. DCF (−5.2 to −8.7) caused down-regulation of the *APOF* gene in all three livers, while DTL (−1.7 to −4.9-fold) down-regulated the gene in two livers and APAP (−2.2-fold) in one liver. Furthermore, the *PON1* (paraoxonase 1) gene is activated by PPAR-γ in the liver and encodes for an enzyme which is a component of high-density lipoprotein. *PON-1* gene expression was down-regulated by DCF (−7.0-fold) and DTL (−2.5-fold), and up-regulated by CBZ (1.8-fold), each in different livers. Moreover, the *ALB* gene encodes for the protein albumin is synthesized in the liver and transports hormones, fatty acids, and other compounds in the serum. *ALB* gene expression was altered by CBZ (−3.8 to 1.4-fold) in all three livers, while DTL (−7.6-fold) exposure down-regulated *ALB* gene expression in one liver.

### 2.9. Heat Shock and ER Stress

Heat shock and ER stress genes were affected across the livers by each drug. In particular, DCF altered 11 of 14 genes (24.5%) in all three livers. The other drugs affected genes in two or one liver, CSA (24 genes, 25.5%) exposure, followed by APAP (15 genes, 16.3%), ETM (12 genes, 18.1%), TBF (9 genes, 13.5%), CBZ (9 genes, 13.5%), DTL (11 genes, 12.3%) and MMI (8 genes, 15%).

Genes encoding for mitochondrial heat shock proteins were altered by DCF, APAP, TBF and CBZ. The *HSPA9* gene encodes for a mitochondrial heat shock protein that plays a role in the control of cell proliferation. Up-regulation of *HSP9A* gene expression occurred with DCF (2.1 to 3.6-fold) in all three livers, while APAP up-regulated *HSP9A* in one liver (1.3-fold). The *HSPD1* gene encodes for a mitochondrial protein essential for the folding and assembly of newly imported proteins in the mitochondria, and may function as a signaling molecule in the innate immune system. *HSPD1* gene expression was altered by TBF (1.8-fold) and by CBZ (−2.1 to 1.9-fold). The *HSPE1* gene encodes for a mitochondrial protein essential in biogenesis, and DTL (−1.8 to −1.6-fold) down-regulated *HSPE1* expression in two livers.

The *HSP90B1* gene encodes for a protein in the endoplasmic reticulum, which plays a critical role in folding proteins, and is an essential immune chaperone to regulate both the innate and adaptive immunity. DCF up-regulated *HSP90B1* (2.0 to 10.9-fold) in all three livers, while CSA caused up-regulation in two livers (1.9 to 6.4-fold), and APAP caused up-regulation in one liver (4.3-fold). ETM (−2.1 to 3.9-fold) altered *HSP90B* expression in two livers. The *HSPB8* gene encodes for a protein superfamily of small heat-shock proteins and functions as a chaperone, involved with macroautophagy, the regulation of cell proliferation and apoptosis. ETM (−2.9 to 3.3-fold) caused changes in *HSPB8* expression in two livers, and CSA (2.6-fold) in one liver. The *CRYAB* gene encodes for a member of the small heat shock proteins (HSP20), α-crystallin B chain protein which functions as a molecular chaperone that binds to misfolded proteins to prevent protein aggregation, as well as inhibit apoptosis and contribute to intracellular architecture. *CRYAB* gene expression was altered by TBF (1.9 to 2.8-fold) and DTL (−1.8 to −3.4-fold) in two livers and by CBZ (−2.9-fold), ETM (3.2-fold) and CSA (−3.8-fold) in one liver. Several genes that encode for members of the DNAJ/HSP40 protein family, and function in a wide range of cellular events, such as protein folding and oligomeric protein complex assembly, were altered. In particular, DCF exposure up-regulated *DNAJC6* (2.9 to 8.6-fold) gene expression in all three livers, and APAP (2.3-fold) in one liver, while DTL down-regulated *DNAJC6* (−2.3 to −3.1-fold) in two livers. APAP also up-regulated *DNAJB6* (1.4 to 2.3-fold) in two livers, and DNAJA2 (1.4-fold) in one liver, and DTL down-regulated *DNAJB1* (−1.5 to −3.9-fold) in two livers. CSA up-regulated *DNAJC3* (2.4-fold) and *DNAJC5* (2-fold) in one liver.

Both the *SEL1L* and *SERP1* genes encode for proteins in the endoplasmic reticulum that have a role in unfolded protein response. The SEL1L protein aids the transport of unfolded proteins from the ER to the cytosol, to be degraded by the proteasome in an ubiquitin-dependent manner. *SERP1* encodes for a stress protein that interacts and protects unfolded target proteins against degradation during ER stress. DCF up-regulated both *SEL1L* (2.5 to 12.8-fold) and *SERP1* (2.4 to 7.1-fold) in all three livers. Additionally, both *SEL1L* and *SERP1* expression were altered in a liver by APAP, MMI, TBF, ETM, and CSA. The *SEC62* gene encodes for an integral membrane protein located in the endoplasmic reticulum, which is part of the SEC61 complex involved with protein translocation apparatus to aid the transport of ER proteins subject to the ubiquitin-proteasome dependent degradation pathway. *SEC62* gene expression was modulated by APAP (−2.6 to 6.4-fold), TBF (−3.4 to 7.9-fold), and CSA (−2.7 to 9.6-fold) in the same two livers, and by CBZ (−4.6-fold), MMI (7.2-fold), and ETM (5.7-fold) in one liver.

Several genes activated encoded for heat shock transcription factors, which activate heat-shock response genes under conditions of stress. Both *ATF4* and *ATF6* genes encode for transcription factors following ER stress. ATF4 belongs to a family of DNA-binding proteins that includes the AP-1 family of transcription factors, the cAMP-response element binding proteins (CREBS), and is involved in protein–protein interactions. ATF6 is embedded in the ER and functions as a stress sensor and transducer of the unfolded protein response. DCF exposure up-regulated *ATF4* (3.3 to 5.1-fold) and *ATF6* (2.3 to 2.8-fold) in all three livers. TBF (2.1-fold) and CSA (1.5-fold) up-regulated *ATF4* in one liver. The *DDIT3* gene encodes for a multifunctional transcription factor, the DNA damage-inducible transcript 3 protein, which induces cell cycle arrest and apoptosis in response to ER stress. DCF up-regulated *DDIT3* (4.9 to 24.8-fold) gene expression in all three livers, while APAP (−1.9-fold) and ETM (−1.8-fold) down-regulated this gene in one liver. The *MBTPS2* gene, (membrane-bound transcription factor site-2 protease) encodes for a member of the intramembrane proteases that cleave several transcription factors involved in the ER stress response and the sterol control of transcription. *MBTPS2* gene expression was up-regulated by APAP (2.4-fold), altered by TBF (−2.4 to 2.4-fold), and down-regulated by CSA (−2.5-fold) and CBZ (−1.5-fold).

Several genes induced by misfolded proteins in the ER (*DERL1*, *EIF2AK3*, *SYVN1*, *HSF2*, *HERPUD1*) were up-regulated by DCF only in this study. The *DERL1* gene (degradation in endoplasmic reticulum protein-1) encodes for a protein that targets misfolded ER proteins for destruction. The *EIF2AK3* gene (eukaryotic translation initiation factor 2-α kinase 3) encodes for an enzyme, located in the ER and induced by misfolded proteins, that phosphorylates and inactivates EIF2 (eukaryotic translation-initiation factor 2), to reduce translational initiation and hence repress protein synthesis. DCF up-regulated the *DERL1* gene (2.8 to 3.8-fold) and the *EIF2AK3* gene (3.4 to 11.5-fold) in all three livers. Furthermore, DCF up-regulated the *SYVN1* (E3 ubiquitin-protein ligase synoviolin) gene, which encodes for an enzyme to remove unfolded proteins accumulated during ER stress by retrograde transport to the cytosol from the ER and via the ubiquitin-proteasome system. DCF up-regulated the *SYVN1* gene (2.2 to 2.5-fold) expression in all three livers. Additionally, *HSF2* encodes for heat shock factor protein 2, which binds to the heat-shock element to activate heat-shock response genes under conditions of stress. The *HERPUD1* gene (homocysteine-responsive endoplasmic reticulum-resident ubiquitin-like domain member 1 protein) encodes for a protein involved in polypeptide folding and in the destruction of misfolded proteins by the ER-associated degradation system. DCF exposure caused an up-regulation of *HSF2* (8-fold) and *HERPUD1* (7-fold) gene expression in the same liver.

### 2.10. Steatosis, Cholestasis, Phospholipidosis

Perturbations in genes associated with steatosis, cholestasis and phospholipidosis were particularly evident across the three livers with APAP (18 genes, 25%), DCF (15 genes, 22.4%), DTL (21 genes, 20.5%) and CSA (17 genes, 19.8%), followed by CBZ (13 genes, 19.5%), TBF (14 genes, 19.1%), ETM (14 genes, 18.1%), and MMI (11 genes, 23.3%).

Several genes for liver transporters were altered in this study, including the ATP-binding sub-families *ABCB1*, *ABCB4*, *ABCC2*, and *ABCC3*, and the solute carriers including *OSTb*, *SLCO1A2*, and *SLC10A1*. The *ABCB1* gene, which encodes for the ATP-dependent membrane efflux pump P-glycoprotein 1 that pumps harmful substances out of the liver cells into the bile ducts, was up-regulated by in all three livers by DCF (2.8 to 4.1-fold), and in one liver by APAP (1.5-fold), TBF (2.2-fold) and CBZ (1.5-fold), and down-regulated by DTL (21-fold). The *ABCB4* gene was down-regulated in all three livers by APAP (−1.5 to −2.2-fold). *ABCC2*, the canalicular multispecific organic anion transporter 1 gene, and *ABCC3*, the canalicular multispecific organic anion transporter 2 gene were both induced by APAP, MMI, TBF, CBZ, and ETM, as a hepatoprotective response. The *OSTΒ* gene, which encodes for the organic solute transporter beta to transport endogenous compounds like taurocholate, prostaglandin E2 across cell membranes, was up-regulated by DCF (5.2-fold) and down-regulated by APAP (−2.7-fold) and ETM (−2.4-fold). The *SLCO1A2* gene that encodes for a sodium independent organic anion transporter of bile acids was down-regulated in three livers by DCF (−7.9 to −28.9-fold), and up-regulated in one liver by MMI (2.1-fold) and CBZ (1.8-fold). The sodium-bile acid cotransporter gene *SLC10A1* was down-regulated in one liver by APAP (−18.3-fold) and TBF (−2.6-fold).

Several of the drugs altered the expression of genes involved with the cholesterol pathway. The *SC4MOL* gene, which encodes for a sterol-C4-methyl oxidase that is involved in the cholesterol synthesis pathway, was altered by APAP (−1.6 to 3.3-fold), TBF (−1.9 to 6.8-fold) and CSA (−2.3 to 6.6-fold) in two livers, and by CBZ (6.4-fold) and ETM (6-fold) in one liver. The cytochrome P450 genes associated with conversion of cholesterol to bile acids, *CYP7A1* and *CYP7B1* were both affected by APAP (−2.8 to 6-fold), ETM (−3.3 to 4.6-fold), DTL (−19.4 to 4.9-fold), CSA (−14.3 to 6.6-fold), TBF (−4.5 to 6.4-fold), and MMI (4.7 to 4.8-fold). DCF affected only CYP7A1 (−51.1-fold) in one liver. Involved in the conjugation and elimination of bile acids is the enzyme encoded by *UGT2B4* (UDP glucuronosyltransferase 2 family, polypeptide B4), which was up-regulated in two livers by APAP (2.1 to 11.4-fold), MMI (2.2 to 4.5-fold), TBF (2.7 to 6.0-fold), and in one liver by ETM (4.1-fold), DTL (5.8-fold) and CSA (4.9-fold).

The alcohol dehydrogenase gene *ADH1C*, and the aldehyde dehydrogenase *ALDH1A1* gene encode for enzymes that facilitate the inter-conversion between alcohols and aldehydes with the reduction of NAD^+^ to NADH. Both genes were down-regulated in all three livers by DCF, and in one liver by DTL. An up-regulation of the *ALDH1A1* gene was detected in two livers with CBZ (1.6 to 2.5-fold) and one liver with APAP (1.9-fold) and MMI (2.2-fold). The *ASNS* gene, asparagine synthetase, encodes for a protein involved in the synthesis of asparagine, a non-essential amino acid produced in the liver. DCF caused up-regulation of the *ASNS* gene expression in all three livers (4.4 to 7.4-fold), while TBF up-regulated it in one liver (2.9-fold).

The *FABP1* (fatty acid-binding protein 1) gene which encodes for a cytoplasmic protein expressed in the liver that binds long-chain fatty acids, fatty acyl CoA, bilirubin, and heme to limit cytotoxicity, was down-regulated in all three livers by APAP (−1.6 to −2.3-fold), in two livers by MMI (−3.5 to −1.7-fold) and DTL (−1.5 to −4.3-fold), and in one liver by DCF (−22-fold) and CBZ (−3.2-fold), while up-regulated in one liver by TBF (7.2-fold) and ETM (5.6-fold). The *LPL* (lipoprotein lipase) gene encodes for a member of the hepatic lipase and endothelial lipase family that hydrolyzes triglycerides in lipoproteins. *LPL* was up-regulated in all three livers by DCF (2.9 to 4.4-fold), and in one liver by APAP (1.9-fold) and ETM (4.5-fold). The *WIPI1* gene encodes for the WD repeat domain phosphoinositide-interacting protein 1, which regulates the assembly of protein and phospholipid interactions, was up-regulated in all three livers by DCF (2.9 to 6.7-fold), and in one liver by CSA (1.8-fold).

DCF was the only drug that altered *FXC1*, mitochondrial import inner membrane translocase, and *MTTP* (microsomal triglyceride transfer protein) gene expression. FXC1 encodes for a protein that mediates the import and insertion of hydrophobic membrane proteins into the mitochondrial inner membrane, and was up-regulated in all three livers (2.5 to 4.0-fold). *MTTP* encodes for a protein involved with lipoprotein assembly and was down-regulated by DCF in all three livers (−2.8 to −11.5-fold).

DTL was the only drug that altered the aquaporin genes *AQP* (−10.9-fold) and *AQP4* (−2.6-fold). These genes encode for integral membrane pore proteins that selectively allow water molecules to go in and out of the cell, while preventing the passage of ions and other solutes.

### 2.11. DNA Damage and Repair

The drugs which altered genes in this category in all three livers included CSA (9 genes, 10.4%), DTL (7 genes, 8.2%), TBF (6 genes, 7.9%), DCF (2 genes, 4.1%), followed by two livers MMI (7 genes, 13.3%), ETM (4 genes, 4.8%), CBZ (2 genes, 2.6%), and in one liver APAP (2 genes, 1.9%). Specifically, two genes *CDKN1* and *ERCC1* were altered by DCF in all three livers. The *CDKN1* and *CDKN1A* genes encode for cyclin-dependent kinase inhibitors, in particular p21 (CDKN1A). DCF up-regulated *CDKN1* in all three livers (2.1 to 3.8-fold) while DTL down-regulated it in two livers. *CDKN1A* was affected only by CSA (−3.2-fold) in one liver. The *ERCC1* gene encodes for the DNA excision repair protein ERCC-1, which is part of an enzyme complex that participates in DNA repair and DNA recombination. DCF up-regulated *ERCC1* in all three livers (2.5 to 24.6-fold), and in one liver by APAP (4.6-fold), MMI (6.7-fold), TBF (6.7-fold), DTL (7.8-fold), ETM (2.5-fold). CSA altered *ERCC1* gene expression levels in two livers (−2.1 to 8.4-fold). The *MSH2* gene encodes for the DNA mismatch repair involved with many types of DNA repair, which was affected in two livers by TBF (−2.6 to 3.5-fold), DTL (−1.7 to 3.2-fold), and CSA (−4.4 to 3.6-fold), and up-regulated in one liver by MMI (3.9-fold) and ETM (3.4-fold).

### 2.12. Apoptosis

Several genes indicative of apoptosis were affected in all three livers by DCF, 6 of 9 genes (14.3%); while drugs which altered the same gene in two livers, included CSA, 4 of 8 genes (11.3%), DTL, 3 of 7 genes (8.2%), TBF, 3 of 4 genes (7.9%), and CBZ, 1 of 6 genes (9.1%). Various apoptotic genes were altered in the three individual livers by APAP (7 genes, 6.7%), and in only two of the livers by ETM, (7 genes, 8.4%) and MMI (4 genes, 6.7%).

The *BID* gene encodes for the BH3 interacting-domain death agonist, which is a pro-apoptotic member of the Bcl-2 family. The *BID* gene expression was up-regulated by APAP (2.8-fold) ETM (2.3-fold) and CBZ (1.4-fold). *BCL2L1* encodes for a BCL-2 member located at the outer mitochondrial membrane that is an apoptosis regulator. CSA altered *BCL2L1* gene expression in two livers. CSA also up-regulated the *APAF1* gene (2.2-fold), apoptotic protease activating factor 1, which encodes a cytoplasmic protein that forms one of the central hubs in the apoptosis regulatory network.

The *FAS* gene encodes for the FAS receptor, which resides on the cell surface to form a death-inducing signaling complex that leads to apoptosis. *FAS* gene expression was up-regulated in the same liver by APAP (5.4-fold), DCF (11-fold), MMI (8.3-fold), TBF (8.0-fold), ETM (5.5-fold), DTL (6.9-fold), and CSA (7.6-fold). The *FADD* gene encodes for the Fas-associated protein with Death Domain, which forms a death inducing signaling complex during apoptosis. FADD gene expression was up-regulated in all three livers by DCF (2.1 to 6.8-fold), in two livers by TBF (1.8 to 2.9-fold) and CSA (2.2 to 2.9-fold), and in one liver by APAP (2.5-fold), MMI (2.7-fold), ETM (2.2-fold), DTL (2.7-fold).

The *TNFRSF10A* and *TNFRSF10B* genes encode for members of the TNF-receptor superfamily involved with apoptosis. DCF up-regulated both these genes in all three livers, while CSA up-regulated *TNFRSF10A* in two livers and CBZ in one liver. The *TNFSF10* gene encodes for a cytokine that is a member of the tumor necrosis factor TNF ligand family, which preferentially induces apoptosis in transformed cells. DCF down-regulated *TNFSF10* gene expression levels in all three livers (−5.0 to −24.8-fold).

Altered *GADD45* gene expression is indicative of growth arrest conditions. DCF up-regulated *GADD45* gene expression levels in all three livers (2.5 to 30.7-fold). The *XIAP* gene encodes for an inhibitor of apoptosis. This gene was up-regulated in all three livers by DCF (2.0 to 5.5-fold), and in the same liver by ETM (3.3-fold), DTL (2.8-fold) and CSA (3.3-fold).

### 2.13. Necrosis

Genes indicative of necrosis were most evident following exposure to APAP (8 genes, 8.7%) and ETM (5 genes, 7.2%), followed by CSA (5 genes, 4.7%), TBF (3 genes, 3.4%), DTL (3 genes, 2.5%), CBZ (1 gene, 1.3%), MMI (1 gene, 1.7%), and DCF (1 gene, 0.7%).

*BMF* encodes for a Bcl-2 protein, which has been associated with apoptosis and necrosis, was up-regulated by ETM (2.2 to 2.8-fold) and CSA (2.2-fold). *NUDT13* (nudix hydrolase 13) gene expression was up-regulated in the same liver by APAP (3.9-fold), MMI (2.6-fold), TBF (3.0-fold), ETM (2.8-fold), DTL (2.9-fold), and CSA (4.8-fold). Furthermore, in this same liver APAP exposure caused the up-regulation of several other genes associated with necrosis, *FOXI1* (2.3-fold), *GALNT5* (2.3-fold), *HOXA3* (2.2-fold), *JPH3* (2.3-fold), *KCNIP1* (2.3-fold), *S100A7A* (2.3-fold).

### 2.14. Immune Response, Inflammation

Genes indicative of inflammation and an immune response exhibited altered expression by most drugs in this study. DCF affected 3 of 4 genes (7.5%) in the three livers. Genes altered in two livers occurred following CSA, 2 of 10 genes (11.3%), ETM, 2 of 7 genes (10.8%), DTL, 1 of 6 genes (5.7%), CBZ, 1 of 7 genes (10.4%), TBF, 1 of 4 genes (5.6%), and MMI 1 of 5 genes (10%). APAP exposure altered 6 genes (5.8%) in two different livers.

*AHSG* (α2-HS glycoprotein) encodes for a plasma binding protein synthesized by hepatocytes. Gene expression of *AHSG* was down-regulated by DCF (−4.2 to −2.0-fold) in all three livers and by MMI (−1.7 to −2.4-fold) in two livers. The interleukin genes exhibited a varied response to the drugs in this study. *IL1Β* gene expression was down-regulated by ETM (−2.5 to −10.2-fold), DTL (−3.5 to −2.4-fold), and CSA (−8.2 to −3.9-fold) in the same two livers, and by MMI (−2.9-fold) in one liver; whereas APAP induced the expression of *IL1A* (2.2-fold) and *IL2* (2.4-fold), CBZ increased *IL4* (56-fold), and CSA increased *IL10* (2.8-fold) gene expression levels. The *C9* gene, which encodes for a member of the complement system, was down-regulated by DCF (−3.8 to −7.6-fold) in the three livers, while up-regulated by CSA (2-fold) in one liver.

*EP300* encodes for a protein that is involved in regulating cell growth, differentiation and division. This gene was up-regulated in the same liver by TBF (2.2-fold), ETM (2.5-fold), DTL (2.2-fold) and CSA (2.5-fold). HRG (histidine-rich glycoprotein) encodes for a glycoprotein produced in the liver and located in the plasma and platelets. *HRG* gene expression was up-regulated by APAP (4.1-fold) in one liver. *PTGS2* (prostaglandin-endoperoxide synthase 2) encodes for the cyclooxygenase 2 (*COX 2*), and the gene expression was up-regulated by DCF (16.9 to 34.8-fold) in two livers, which is tied to the pharmacological mechanism of DCF.

## 3. Discussion

The goal of this study was to characterize human response ex vivo to drugs associated with liver side-effects clinically. In this study, functional markers of oxidative stress plus gene expression profiles indicative of organelle and tissue dysfunction differentiated the drug induced tissue stresses across drugs in the same human liver tissue. These drugs demonstrated diverse effects and can serve as reference drugs to evaluate the safety risks of drugs in development. The ex vivo model used in this study, organotypic human liver slices, has the leverage as a relevant model since cell-cell and cell-matrix interactions are represented in their normal architecture to mimic in vivo dynamics to forecast drug safety risks. The doses of the drugs were selected either from the literature citing plasma concentrations associated with clinical side-effects or from previous in vitro human liver preparations or from human liver slice studies in which the concentration exhibited a change in tissue function [8,30,32]. For example, high doses of the pain medication APAP (4 g dose or blood levels of 1 mM) are associated with altered liver function in some healthy males [28]. All drugs were tested side-by-side in each human liver, and the drugs were dosed daily to mimic initial dosing in clinical studies.

Several drugs in this study are known to perturb tissue anti-oxidant status, and the metabolism of the drug contributes to oxidative stress, mitochondrial injury, and ER stress in some individuals. For example, the acetaminophen metabolite *N*-acetyl-p-benzoquinone imine (NAPQI) is conjugated with GSH, and other metabolites formed by peroxidase-like enzymes can reduce the liver anti-oxidant status [33]. APAP metabolite profiles have been shown to distinguish responders from non-responders toward APAP hepatotoxicity [34]. Mitochondria are a key target of APAP due to a reduction of mitochondrial GSH levels by ROS, leading to necrotic cell death [35,36,37,38]. Diclofenac, a non-steroidal anti-inflammatory agent, generates reactive metabolites that bind to cellular macromolecules and proteins to alter tissue anti-oxidant status. Mitochondrial injury is proving to be a key factor in DCF hepatotoxicity, and an immune mediated hypersensitivity is seen in some individuals [18,39,40]. Methimazole, an agent to decrease thyroid size, and linked with hematologic effects and hepatoxicity, co-oxidizes GSH to GSSG via the metabolism of the thione moiety [41]. The reduced GSH tissue contributes to subsequent toxic effects [42,43,44,45]. Clinically, a reduction of the MMI dose from 30 to 15 mg/day reduces the incidence of side-effects [46]. Two additional compounds that could affect the tissue anti-oxidant status as a consequence of metabolism are terbinafine, an allylamine derivative used as an antifungal agent, and carbamazepine, an iminostilbene used for seizures. Both drugs likely generate reactive metabolites because hepatotoxicity, detectable by increases in serum transaminases, is associated with hypersensitivity or an immunological response in some individuals, yet evidence for adduct formation has not been reported [29,30,47,48].

Pharmaceutical agents that modulate mitochondria function raise concern about the potential for mitochondrial injury. Drugs in this study associated with a direct mitochondrial interaction include etomoxir considered for diabetes and heart failure, the immunosuppressant cyclosporine, and dantrolene a smooth muscle relaxant. ETM irreversibly inhibits the rate-limiting enzyme of mitochondrial β-oxidation, carnitine palmitoyltransferase-1 (CPT-1), to decrease the use of free fatty acids as a source of energy so as to increase the utilization of glucose for ATP production [49]. CSA inhibits the mitochondrial permeability transition pore through binding to a regulator of the pore cyclophilin D. High CSA concentrations are associated with mitochondrial injury [1,50,51,52,53,54]. Dantrolene, a potential muscle relaxant, affects Ca^+2^ homeostasis via ryanodine receptors. The hepatic injury may in part be due to a perturbation of Ca^+2^ homeostasis or the formation of reactive oxygen species [55]. The RyR1 receptor is located on the inner mitochondrial membrane of excitable cells and is postulated to exist within liver mitochondria [1]. Hepatocytes also possess a truncated type 1 ryanodine (RYR1) receptor in the endoplasmic reticulum [56]. Both CSA and DTL at low doses can influence calcium homeostasis to minimize apoptosis or necrosis during ischemia-reperfusion injury [57,58].

Functional tissue biomarkers of oxidative stress used in this study were liver slice ATP and GSH levels following drug exposure. ATP is a sensitive marker of oxidative stress and an indicator of mitochondrial function. GSH, the major anti-oxidant synthesized in the liver, is an indicator of overall tissue anti-oxidant status. About 15% of the cellular GSH resides within the mitochondria, and mitochondria are a major source of ROS production, which drugs can disrupt to impact mitochondrial function and cause injury [1,40,59,60]. Both APAP and DCF caused significant time-dependent decreases of ATP levels, and DTL caused significant decreases at 72 h. GSH levels were significantly increased with APAP, MMI and CBZ followed by TBF, DTL, and ETM. Since the metabolism of these drugs consumes GSH (APAP, MMI, CBZ and TBF) or affects the mitochondria directly (DTL, ETM), the tissue likely responds by synthesizing GSH. Previous human liver slice studies using several concentrations per drug revealed that 1 mM APAP, 500 μM MMI, 10 μM CSA, and 10 μM DTL altered ATP or GSH levels in most but not all human liver slice studies [8,27].

### 3.1. Metabolism Genes

The induction of cytochrome P450s by xenobiotics can have a protective effect, and yet may trigger deleterious effects. For example, POR, the cytochrome p450 oxidoreductase, facilitates electron transfer from NADPH to all microsomal P450 enzymes. The gene expression of *POR* was altered by DCF and TBF. If this were to result in an increased respiration rate, it could further increase the formation of ROS within mitochondria to perturb Ca^+2^ homeostasis and cell signaling [1,35,36]. Furthermore, mitochondrial DNA, lipids and proteins are important targets of ROS [61]. The cytochromes CYP1A1 and CYP1A2, inducible by xenobiotics, interact with mitochondria and may be linked with trans-membrane potential and apoptosis [62]. In this study, TBF, MMI, DTL, APAP, CBZ, and DCF caused changes in the *CYP1A* gene expression. DCF and DTL also affected *FMO* gene expression levels. Induction of FMO enzymes in mice is protective toward APAP hepatotoxicity [63], whereas inhibition of FMO suppressed MMI hepatotoxicity [42].

### 3.2. Oxidative Stress, Mitochondrial Energy, Heat Shock and ER Stress, Apoptosis, Necrosis, DNA Damage and Repair

The APAP and DCF induced decreases of ATP levels and changes in GSH levels were paralleled by gene expression changes of oxidative stress (10.6% APAP, 7.5% DCF) and mitochondrial energy (6.7% APAP, 2.0% DCF). In particular, the oxidative stress genes affected by both drugs included glutathione regulation (*GSTA3*, *GSTA4*), reactive intermediate formation (*GPXs*, *EPHX1*), and a regulator of translation (PPP1R15B). Mitochondrial energy genes altered by APAP included *MDH1* (malate dehydrogenase), which is part of the TCA cycle, and the mitochondrial matrix succinyl-CoA ligase (*SUCLG1*), while DCF altered a mitochondrial uncoupling protein (*UCP2*), which can separate oxidative phosphorylation from ATP synthesis. Other studies report that mitochondrial uptake of DCF via the anion carrier results in uncoupling of respiration and opening of the permeability transition pore to initiate cell death [39]. Additionally, the binding of DCF reactive electrophiles to mitochondrial proteins triggers apoptosis [64] and possibly a hypersensitivity reaction [65].

An increase of unfolded proteins in the endoplasmic reticulum (ER) triggers a stress response, which includes the increased expression of proteins involved in polypeptide folding, the inhibition of translation to prevent further accumulation of unfolded proteins, the destruction of misfolded proteins, and an increased transcriptional regulation of protein synthesis leading to cell death [3,66]. The ER is the major intracellular storage for Ca^+2^ and interacts with mitochondria to exchange metabolic signals via lipids, proteins, metabolites and Ca^+2^. Mitochondrial Ca^+2^ overload triggers the mitochondrial pore to release mediators including Ca^+2^, cytochrome c, and pro-apoptotic proteins [38,61,67,68,69,70].

In this study, both APAP and DCF altered the expression of a mitochondrial heat shock gene (*HSP9A*), the protein folding genes (*DNAJ* family), and genes encoding for the transport of unfolded proteins to the proteasome for degradation (*SEL1L*, *SERP1*). DCF caused changes in several genes induced by misfolded proteins in the ER (*DERL1*, *EIF2AK3*, *STVN1*, *HSF2*, *HERPUD1*), and several genes that encode for heat shock transcription factors (*ATF4*, *ATF6*, *DDIT3*). Consequences of ER and mitochondria stress can trigger various cell types to release pro-inflammatory or cytotoxic mediators to activate cell death signaling pathways. APAP induced a greater proportion of genes linked with necrosis (8.7%), while DCF induced gene changes linked with apoptosis (14.3%). Genes for the death receptor FAS (*FAS*, *FADD*), associated with necrosis [59], were up-regulated by APAP and DCF. Additionally, DCF up-regulated *GADD45*, a gene associated with growth arrest, and genes of the TSF-receptor family (*TNFRS10A and B*) involved with apoptosis. Marked decreases of liver GSH have been demonstrated to sensitize hepatocytes to the oxidative effects of cytokines such as tumor necrosis factor [66,71]. APAP caused the up-regulation of several genes linked with necrosis (*FOXl1*, *GALNT5*, *HOXA3*, *JPH3*, *KCNIP1*, *NUDT13*, *and S100A7A*). The concentrations of APAP (1 mM) and DCF (1 mM), which induced these changes are considered high, however such APAP concentrations have been tested in humans. The FDA is requiring additional labeling for APAP reminding consumers to be mindful of the dose taken [72]. Acetaminophen is the primary choice for pain in US hospitals, and is a concern for the elderly, which likely have a compromised liver GSH status.

Both TBF and CBZ (100 μM) increased liver slice GSH levels and had an impact on oxidative stress (18% TBF, 11.7% CBZ) and mitochondrial energy gene expression (4.5% TBF, 7.8% CBZ). In particular, both drugs affected the expression of glutathione transferase *GSTA3*, gene markers of reactive intermediate formation with glutathione peroxidase isoforms (*GPX2 and 3*), microsomal epoxide hydrolase 1 (*EPHX1*), and reactive oxygen formation (*DUOX2*). TBF additionally up-regulated the *NQO1* gene expression, involved in the reduction of quinones to hyroquinones, and the translation factor *PPP1R15B*. Genes involved with the TCA cycle and cellular energy, aconitase (*ACO1*) and malate dehydrogenase (*MDH1*), were altered by TBF and CBZ. Additionally, CBZ affected the mitochondrial gene expression of succinate dehydrogenase (*SDHC*), succinyl CoA ligase, and dihydrolipoamide dehydrogenase (*DLD*) involved in multi-enzyme complexes associated with energy metabolism, while TBF affected the mitochondrial cytochrome C1 (*CYC1*) gene expression. Drug induced tissue stress reflected by changes of heat shock and ER stress genes represented about 13% for both drugs. In particular, a mitochondrial heat shock gene (*HSPD1*) indicative of protein folding of imported proteins, ER protein folding chaperone genes (*HSP90B1*, *CRYAB*), unfolded protein response genes (*SEL1L*, *SERP1*, *SEC62*), and the membrane transcription factor *MBTPS2* gene. Genes indicative of apoptosis represented 7.9% for TBF and 9.1% for CBZ, and necrosis gene changes represented 3.4% with TBF and 1.3% for CBZ. The apoptosis genes induced by TBF included the FAS death pathway and *caspase 8*, while CBZ affected *GADD45*. Differences between the two drugs became evident with a greater change of genes linked with regulation for TBF (7.9%) than for CBZ (1.3%). In particular, TBF altered the mismatch repair gene (*MSH2*), and the excision repair genes (*ERCC1 and 6*).

MMI (500 μM) exposure altered the fewest oxidative stress (6.7%) genes, yet important gene indicators of oxidative stress. Two genes that combat oxidative stress were up-regulated by MMI, *NQO1* involved in the reduction quinones to hydroquinones, and *PRDX1* which reduces hydrogen peroxide and alkyl hydroperoxides. Of particular interest for MMI is that Kupffer cells contain myeloperoxidases, which contribute to its metabolism and co-oxidation of GSH. In this study, the utilization of GSH was compensated for by an increase of liver slice GSH levels, and hence no effect on GSH utilization genes in this study. In a previous human liver-blood co-culture study, liver slice GSH levels were decreased by MMI exposure at 72 h, which is likely due to the overall liver GSH status being lower than in this study. Blood cells revealed a decline of GSH levels due to MMI metabolism prior to a decline of liver GSH levels [25]. MMI also affected mitochondrial energy genes (6.7%), including *MDH1* and *CYC1*, that corresponded to decreased ATP levels at 72 h. MMI induced heat shock gene expression changes (15%) involved with the ubiquitin pathway for protein degradation (*UBE2G2*, *SEC62*, *SEL1L*, and *SERP1*). Genes linked with apoptosis (6.7%) and necrosis (1.7%) were altered the least in comparison to the other drugs. However, the proportion of genes indicative of DNA damage and repair (13.3%) represented the greatest change compared to the other drugs. In particular, the gene changes dealt with DNA repair, *BRCA2*, *ERCC1* and *MSH2*.

The drugs known to interact directly with mitochondria DTL, ETM, and CSA trigged oxidative stress (7.4% DTL, 10.8% ETM, 8.5% CSA) and mitochondrial energy (3.3% DTL, 6% ETM, 2.8% CSA) gene expression changes. Glutathione regulation genes were affected, such as *GSTA3* (ETM), and *GPX2* (DTL, CSA), as well as genes linked to reactive oxygen formation, *DUOX1* and *DUOX2* (CSA). Heat shock and ER stress related genes were greatly affected by CSA (25.5%), followed by ETM (18.1%) and DTL (12.3%). *HSP90B1* gene expression, involved with protein folding and as an immune chaperone, was altered in two livers by CSA and ETM. Consequences for apoptosis gene expression changes represented 11.3% CSA, 8.4% ETM and 8.2% DTL, and for necrosis represented 4.7% CSA, 7.2% ETM, and 2.5% DTL. The *FADD* and *FAS* genes, linked with the apoptotic death pathway, were perturbed by CSA, DTL and ETM, as well as *TNFRSF10A* by CSA.

### 3.3. Fatty Acid Metabolism, Steatosis, Cholestasis, Phospholipidosis, Immune Response and Inflammation

The perturbation of fatty acid metabolism can have consequences on the genes associated with steatosis, cholestasis, and phospholipidosis. The combined categories were greatest for DTL (45%), followed by APAP (34.6%), DCF (30.6%), ETM (32.6%), CBZ (33.8%), TBF (29.2%), MMI (26.5% MMI), and CSA (25.6%) of the total gene expression changes. Gene changes in at least two livers were greater for DCF (9 of 12 genes) and APAP (6 of 8 genes) for the steatosis, cholestasis, phosopholipidosis category; whereas DTL altered more genes in two livers for fatty acid metabolism (7 of 12 genes). Several liver transporter genes exhibited altered expression, in particular the efflux p-glycoprotein transporter *ABCB1* (DCF, APAP, DTL, CBZ), the cannicular organic anion transporter 1 and 2, *ABCC2* and *ABCC3*, (APAP, MMI, TBF, CBZ and ETM), the organic solute transporter gene *OSTβ* (DCF, APAP, ETM), the organic anion transporter *SLCO1A2* (DCF, MMI, CBZ), and the sodium-bile acid co-transporter *SLC10A1* (APAP, TBF). The perturbation of several transporter genes indicates that the tissue is ramping up the export of bile acids and solutes to prevent cholestatic hepatotoxicity [73].

Genes associated with the cholesterol synthesis, *SC4MOL* (APAP, TBF, CSA, CBZ, ETM), the conversion of cholesterol to bile acids *CYP7A1* and *CYP7B1* (APAP, ETM, DTL, CSA, TBF, MMI and DCF), and the elimination of bile acids *UGT2B4* (APAP, MMI, TBF, ETM, DTL, CSA) exhibited altered expression. The *FABP1* gene, which encodes for a protein that binds long chain fatty acids, acyl CoA, bilirubin and heme to limit cytotoxicity was affected by all drugs except CSA. Cyclooxygenase 2 (*PTGS2*) gene expression was affected by DCF, which is in line with the pharmacological mechanism of DCF inhibiting this enzyme. Various interleukin genes were altered including *IL1β* (ETM, DTL, CSA, MMI), *IL1A* and *IL2* (APAP), *IL4* (CBZ), *IL10* (CSA), *C9* (DCF, CSA), suggestive of an inflammation and possible immune response. Proinflammatory and cytotoxic mediators released from macrophages, may contribute to APAP hepatotoxicity in some individuals [74].

Drugs that influence fatty acid metabolism can alter both mitochondrial function and peroxisome function to affect the overall energy levels of the tissue. Drug effects on mitochondrial genes are of particular concern since mitochondrial toxicity could become an issue. Several mitochondrial genes (*CPT1*, *ACADM*, *ACAA1*, *ACADVL*, *ACAA2*, *HADHA*, *ECHS1*, *HADHB*) involved in the β-oxidation pathway of fatty acids were altered by ETM (6 genes), DTL (6 genes), TBF (3 genes), CBZ (3 genes), CSA (3 genes) and APAP (2 genes). Neither DCF nor MMI influenced these mitochondrial genes. DCF affected only a mitochondrial inner translocase gene *FXC1*. Severe or chronic impairment of mitochondrial β-oxidation free fatty acid (FFA) metabolism can lead to liver lipid deposits, microvesicular steatosis and inflammation, and necrosis in rats and man [75,76]. Hepatotoxicity is attributed to interference of the respiratory complexes, decreased ATP production, and oxidative stress [75,77,78,79]. Direct inhibitors of β-oxidation (e.g., tetracyclines, phenformin, troglitazone) or drugs that sequester CoA (e.g., valproic acid) to inhibit β-oxidation have caused dicarboxylic aciduria, and lactic acidosis [78,79]. In humans, drug induced lactic acidosis can lead to hepatic failure, and is correlated with enlarged mitochondria [80]. In previous liver slice studies, compounds that inhibited CPT1 or which complexed with CoA to limit the capacity of the β-oxidation pathway led to mitochondrial injury at high doses. In rats the mitochondria were enlarged both in vivo and in vitro, and in human liver slices the mitochondria exhibited granular inclusions [81,82].

In this study, 8 drugs associated with liver effects clinically were compared side-by-side in individual human liver slice experiments. ATP and GSH levels following drug exposure were sensitive functional markers of oxidative stress and mitochondria dysfunction. Gene expression profiles indicative of organ dysfunction and injury further characterized and distinguished drug effects (Table 5). Each drug had direct effects on the livers and caused multifactorial effects, for which consequences on mitochondrial energy, ER calcium regulation, cholestasis, and inflammation, will be underlying mechanisms of the clinical effects. Activation of oxidative stress, mitochondrial energy, heat shock and ER stress genes was particularly evident with CSA (37%), TBF (36%), ETM (35%), APAP (34%), DCF (34%) and CBZ (33%). Adding DNA damage and repair, apoptosis, necrosis, inflammation and immune response revealed CSA (75%) with the greatest effect followed by ETM (66%), TBF (61%), DCF (61%), MMI (60%), APAP (57%), CBZ (56%), and DTL (48%). Gene changes in fatty acid metabolism, steatosis, cholestasis plus immune response and inflammation were affected by DTL (51%), followed by CBZ and ETM (44%–43%), APAP and DCF (40%–38%), then MMI, TBF and CSA (37%–35%).

A comparison of drugs in development to reference drugs aids in drug candidate selection with the widest safety margin, and the identification of serum concentrations that would lead to organ injury, if such concentrations were achieved. Drug specific safety biomarkers can be identified for clinical studies, which may include formation of a metabolite, mitochondrial injury markers, or indicators of cholestasis, inflammation and immune response markers. Some of the drugs in this study are also linked with hypersensitivity reactions. As more studies are done early markers of hypersensitivity will be identified. To improve the objectivity and semiquantitation to assess drug induced liver injury (DILI) and clinical assessments the Roussel Uclaf Causality Assessment Method (RUCAM) is showing success particularly with hypersensitivity cases [83,84]. Further validation is needed, as well as reliable assessments for hepatitis or cholestasis [84]. The multifactorial nature of drug induced liver injury and idiosyncratic-DILI requires continued approaches of multiparametric endpoints including drug metabolism enzymes, antioxidant enzymes, drug transporters, and inflammation plus modeling to identify predictive assays and endpoints for diagnosing individual drug-induced liver injury [85,86].

## 4. Materials and Methods

### 4.1. Chemicals and Reagents

Acetaminophen (cat #A7085), carbamazepine (cat #C8981), cyclosporin A (cat # 30024), dantrolene sodium salt (cat #D9175), diclofenac sodium salt (cat #PHR1144), (+)-etomoxir sodium salt hydrate (cat #E1905), methimazole (cat #M8506) and terbinafine hydrochloride (cat #T8826) were purchased from Sigma-Aldrich (St. Louis, MO, USA).

Waymouth’s MB 752/1 (without l-glutamine, phenol red and sodium bicarbonate), and fetal bovine serum were purchased from Invitrogen (Chicago, IL, USA). l-glutamine, Antibiotic/Antimycotic solution and gentamicin sulfate were obtained from Sigma-Aldrich (St. Louis, MO, USA). V‑7 preservation solution was provided by Vitron, Inc. [87]. Mixed cellulose‑ester IMMOBILIN‑NC filters (HATF, 0.45 µm surfactant and triton free, autoclavable) used to support the liver slices were obtained from Millipore (Bedford, MA, USA). Dithiobis-nitrobenzoic acid, reduced GSH luciferin, luciferase, and ATP were purchased from Sigma-Aldrich.

### 4.2. Biologicals

Human tissue was procured with donor consent using procedures approved by medical and ethical standards through IIAM (Edison, NJ, USA). Extensive serologies demonstrated the absence of many infectious agents including HIV and hepatitis. The tissue is of transplantation grade. It is explanted from the donor, and perfused with Viaspan^®^, under protocols used for organ transplantation. Reasons for the occasional availability of these tissues for research include anatomical irregularities, histological findings, age of donor, time in transit, or status of the recipient. The liver was transported in Viaspan^®^ on ice.

### 4.3. Human Liver Slice Cultures

Upon arrival of the liver to the Vitron laboratory, it is immediately cored (8 mm diameter cores) and precision‑cut slices prepared (200 ± 25 µm thick) in oxygenated V‑7 cold preservation solution with the Brendel/Vitron tissue slicer (Vitron Inc., Tucson, AZ, USA). Each slice was floated onto a sterile Immobilon‑NC transfer membrane and placed on a titanium roller insert (Vitron Inc.). The rollers were blotted and placed into 20 mL glass scintillation vials containing 1.7 mL of Waymouth’s MB 752/1 tissue culture medium, fortified with 2.24 g/L sodium bicarbonate, 0.35 g/L l‑glutamine, 10 mL/L Antibiotic/Antimycotic solution, 84 µg/mL gentamicin sulfate and 10% fetal bovine serum. The human liver slices were incubated in a Dynamic Organ Culture Incubator (Vitron Inc.), rotating at 1 rpm and maintained at 37 °C in a 95% O_2_:5% CO_2_ atmosphere.

The human liver slices were exposed to either acetaminophen (APAP 1 mM), cyclosporin A (CSA 10 μM), diclofenac (DCF 1 mM), dantrolene (DTL 10 μM), etomoxir (ETM 100 μM), methimazole (MMI 500 μM), terbinafine (TBF 100 μM), or carbamazepine (CBZ 100 μM) as a final concentration in the culture medium which was dispensed into the vials. The vehicle concentration in the cultures was 0.1% DMSO, which does not alter human liver slice function compared to untreated human liver slices [8]. Daily dosing of drug treatment was achieved by replacement of the medium at 24 and 48 h. For the functional assays (ATP and GSH at 24, 48, 72 h) and gene expression (48 h) evaluation, 6 treated human liver slices and 10 vehicle control slices were collected for each drug at each time-point and for each liver (*n* = 3).

### 4.4. Functional Assays

Human liver slices collected for ATP and GSH assays were weighed, placed in 1.0 mL 10% trichloroacetic acid (TCA), disrupted (~5 s) with a Power Gen 125 homogenizer (Fisher Scientific, Pittsburgh, PA, USA), snap frozen in liquid nitrogen, and stored at −76 °C until analysis. ATP content was determined on human liver slice homogenate supernatants, following a 10,000× *g* centrifugation for 5 min at 4 °C. Samples (5 µL) were diluted with 1 mL HEPES (25 mM) and 200 µL was then added to a 96 well microtiter plate. Standards were prepared in 10% TCA, diluted with HEPES, and 200 µL added per well in duplicate. The ATP standard curve was from 0–200 µM. The Luciferin‑Luciferase solution (Luciferase 2 µg/mL, Luciferin 50 µM (5%), stabilizing buffer (50%), 25 mM HEPES buffer (45%)) was injected at 100 µL/well and luminescence was read using a Tropix TR717 Microplate Luminometer (Applied Biosystems, Bedford, MA, USA). Data for the liver slice is presented as nmols/mg wet weight. A one-way ANOVA followed by a Dunnett’s post-test was performed comparing the treated versus time-matched control values using GraphPad Prism software version 5.0 (GraphPad Software Inc., San Diego, CA, USA).

GSH content was determined on liver slice homogenate supernatants (50 µL), following a 10,000× *g* centrifugation for 5 min at 4 °C, and transferred to a 96 well microtiter plate. Ellman’s reagent (200 µL; 39.6 mg dithiobis‑nitrobenzoic acid/10 mL EtOH diluted 1:10 with 0.5 M Tris‑1 mM EDTA buffer, pH 8.9) was added. The absorbance was determined at 405 nm using a Titertek Multiskan MCC/340. The values were compared to a standard curve of reduced glutathione (0–250 µM), and the data is presented as nmols GSH/mg slice wet weight for the liver slices. Standards were prepared and added at 50 µL/well in duplicate. A one-way ANOVA followed by a Dunnett’s post-test was performed on the treated versus time-matched control values using GraphPad Prism software version 5.0 (GraphPad Software Inc.).

K^+^ retention was determined using a NOVA 1 electrolyte analyzer (NOVA Biomedical, Boston, MA, USA). Liver slices at 1, 4, 24, 48, 72 h (4 slices/time-point) were disrupted by sonication in 250 µL distilled water and centrifuged (10,000× *g* for 5 min). The resulting supernatant was analyzed for K^+^ content. Results are expressed as µmols K^+^/g slice wet weight.

### 4.5. Gene Expression

Human liver slices were placed into RNAse free 2.0 mL microfuge tubes, frozen immediately in liquid nitrogen, and stored at −76 °C. Total RNA was isolated using Qiagen RNeasy mini kits with a trizol chloroform extraction by SABiosciences (Frederick, MD, USA). RNA samples were assayed for quality using the Agilent 2100 Bioanalyzer (Agilent Technologies, Inc., Foster City, CA, USA), and for yield using the NanoDrop spectrophotometer (Thermo Scientific, Inc. (Waltham WA, USA). RNA with an integrity number (RIN) ≥6.0 was used for amplification.

PCR arrays (SABiosciences) employ qRT-PCR and examine focused sets of genes. In this study, the human specific molecular toxicology pathway finder PCR array (PANZ-3401, 370 genes) was run at SABiosciences. For vehicle control and drug treated samples, cycle threshold (*C*_t_) values were obtained and normalized to *C*_t_ values of the housekeeping genes (Actb, B2M, GAPDH, Hprt-1, Rplp-0). The qRT-PCR array results for each gene was compiled as average fold change of drug treated versus vehicle treated liver slices and *p*-values by SABiosciences.

Gene ranking is comprised of different gene expression parameters, such as fold change and its *p*-value, into one meta-characteristic, and the algorithm is described in detail elsewhere [88]. Gene ranking identified the gene changes with a false discovery rate (FDR) of 15% or less. For statistical analysis two parameters were selected: gene expression fold change and *t*-test probability of gene expression change. The gene expression fold change rank was calculated based on a ratio of average log_2_ intensities of gene *g* in drug treated and vehicle treated liver slices. A two-tailed Student’s *t*-test (*p*-value ≤ 0.05) has been used to estimate significance between gene expression fold-change in drug treated and vehicle treated slices.

## 5. Conclusions

In this study eight drugs known to exhibit liver effects clinically were compared side-by-side in individual human liver experiments and dosed daily. Functional markers of oxidative stress and mitochondria dysfunction, liver GSH and ATP levels, revealed both increased levels following drug exposure due to a compensatory response of tissue consumption and decreased levels when the tissue could no longer maintain homeostasis. Gene expression of categories linked with altered tissue function and injury revealed that the drugs had multifactorial effects on human liver. Changes in oxidative stress, ER stress, mitochondria, and fatty acid metabolism progressed to changes in apoptosis, necrosis, cholestasis, inflammation and immune response to reveal the underlying mechanisms of liver effects. Comparing these reference drugs with drugs in development aids in decisions on safety and could guide biomarker selection for clinical studies. 

## Figures and Tables

**Table 1:** Human donor information and markers of human liver slice viability (K^+^, ATP and GSH levels) in control slices (24–72 h) to verify the quality of the tissue for the duration of the experiment.

Donor	Age/Sex/Race	Medications	Cold Ischemia (h)	K^+^ μmols/gm Wet Weight 24, 48, 72 h	ATP nmols/mg Wet Weight 24, 48, 72 h	GSH nmols/mg Wet Weight 24, 48, 72 h
HL869	68/M/C	none	19	83.6, 93.7, 94.1	15.2, 16.5, 18.6	8.9, 11.3, 10.9
HL870	21/M/C	Prednisone	14	80.4, 83.4, 87.7	15.0, 13.0, 11.2	10.6, 13.4, 14.6
HL871	28/F/A	none	16	78.9, 89.2, 87.4	13.5, 15.4, 15.5	16.5, 16.5, 14.3

M, Male; F, Female; C, Caucasian; A, Asian.

**Table 2:** Mean ATP and GSH values represented as a decimal from time-matched control human liver slices at 24, 48 and 72 h of culture in the presence of various drugs. Statistical significance (*) was determined using the Holm Sidak method of multiple *t*-tests with α = 5% (Prism, GraphPad 6.0).

**% Change**	**Mean ATP, 24 h HL869 HL870 HL871**			**24 h**	**Mean ATP, 48 h HL869 HL870 HL871**			**48 h**	**Mean ATP, 72 h HL869 HL870 HL871**			**72 h**
APAP	1.04	0.89	0.99	0.97	0.91	0.80	0.83	0.85 *	0.84	0.74	0.75	0.78 *
DCF	0.48	0.80	0.63	0.63 *	0.55	0.50	0.55	0.53 *	0.49	0.18	0.15	0.27 *
MMI	0.98	0.83	1.07	0.96	0.93	1.10	1.06	1.03	0.96	0.86	0.67	0.83
ETM	0.96	1.12	0.65	0.91	0.71	1.27	1.00	0.99	0.79	0.80	0.97	0.85
DTL	0.68	1.02	1.41	1.03	0.88	1.08	1.15	1.03	0.82	0.82	0.78	0.81 *
CSA	1.00	0.96	1.13	1.03	0.74	0.69	1.07	0.83	0.70	1.14	0.83	0.89
TBF	0.86	0.85	1.14	0.95	0.87	1.00	1.07	0.98	0.75	1.30	1.04	1.03
CBZ	1.01	0.78	0.77	0.85	0.96	1.02	1.11	1.03	0.83	1.01	1.06	0.97
**% Change**	**Mean GSH, 24 h HL869 HL870 HL871**			**24 h**	**Mean GSH, 48 h HL869 HL870 HL871**			**48 h**	**Mean GSH, 72 h HL869 HL870 HL871**			**72 h**
APAP	1.24	1.25	1.03	1.17	1.15	1.02	1.04	1.07	1.35	1.21	1.39	1.32 *
DCF	0.71	0.73	0.52	0.65 *	0.91	0.85	0.58	0.78	0.95	0.70	0.44	0.70
MMI	1.15	1.37	0.99	1.17	1.11	1.49	1.32	1.31	1.30	1.49	1.31	1.37 *
ETM	1.02	1.16	0.76	0.98	0.97	1.32	1.02	1.10	1.00	1.24	1.13	1.12
DTL	0.80	1.25	1.22	1.09	0.77	0.98	1.25	1.00	0.93	1.31	1.68	1.31
CSA	1.06	1.09	0.94	1.03	0.68	0.90	0.98	0.85	0.72	0.94	1.07	0.91
TBF	0.98	1.22	0.98	1.06	1.09	1.22	1.02	1.11	1.07	1.21	1.39	1.22
CBZ	1.15	1.16	0.65	0.99	1.17	1.35	1.21	1.24 *	1.19	1.18	1.29	1.22 *

**Table 3:** Number of significant gene expression changes per category representative of toxicological pathways for each drug and each human liver experiment.

**Gene Categories**	**APAP**			**DCF**			**MMI**			**TBF**		
	**HL869**	**HL870**	**HL871**	**HL869**	**HL870**	**HL871**	**HL869**	**HL870**	**HL871**	**HL869**	**HL870**	**HL871**
Metabolism	3	3	3	5	3	5	2	4	2	4	2	3
Oxidative Stress	3	4	4	4	4	3	0	1	3	7	5	4
Mitochondria Energy	2	3	2	1	1	1	0	3	1	0	3	1
Heat Shock and ER Stress	6	4	7	11	11	14	1	4	4	4	4	4
Fatty Acid Metabolism	4	3	3	6	3	3	0	2	0	5	3	1
Steatosis, Cholestasis, Phospholipidosis	8	9	9	14	9	10	1	10	3	12	3	2
DNA damage, repair	0	0	2	2	2	2	0	5	3	1	3	3
Apoptosis	1	1	5	7	6	8	0	2	2	2	3	2
Necrosis	0	2	7	0	0	1	0	0	1	2	0	1
Immune Response, Inflammation	0	2	4	3	4	4	0	4	2	1	1	3
Total	27	31	46	52	43	51	4	35	21	34	27	24
**Gene Categories**	**CBZ**			**ETM**			**DTL**			**CSA**		
	**HL869**	**HL870**	**HL871**	**HL869**	**HL870**	**HL871**	**HL869**	**HL870**	**HL871**	**HL869**	**HL870**	**HL871**
Metabolism	4	3	1	1	0	0	3	4	2	0	1	1
Oxidative Stress	5	4	0	1	2	6	6	0	3	3	3	3
Mitochondria Energy	0	2	4	2	2	1	2	2	0	1	2	0
Heat Shock and ER Stress	3	5	2	2	5	8	5	9	1	11	12	4
Fatty Acid Metabolism	3	4	4	5	5	2	21	1	8	2	0	2
Steatosis, Cholestasis, Phospholipidosis	7	4	4	8	4	3	11	4	10	9	8	4
DNA damage, repair	0	1	1	0	1	3	4	2	4	3	3	5
Apoptosis	2	1	4	0	1	6	4	2	4	5	2	5
Necrosis	0	0	1	1	3	2	1	1	1	2	2	1
Immune Response, Inflammation	0	3	5	1	4	4	1	3	3	4	4	4
Total	24	27	26	21	27	35	58	28	36	40	37	29

**Table 4:** Significant gene expression changes in human liver slices exposed daily for 48 h to APAP (1 mM), DCF (1 mM), MMI (500 μM), TBF (100 μM), CBZ (100 μM), ETM (100 μM), DTL (10 μM), CSA (10 μM). Values represent the fold change for the highest ranked genes (FDR = 15%) in human liver slices (*N* = 3 donors, *n* = 10 control and 6 treated slices/drug/donor).

Genes	APAP			DCF			MMI			TBF			CBZ			ETM			DTL			CSA		
	HL869	HL870	HL871	HL869	HL870	HL871	HL869	HL870	HL871	HL869	HL870	HL871	HL869	HL870	HL871	HL869	HL870	HL871	HL869	HL870	HL871	HL869	HL870	HL871
**Metabolism**																								
POR				2.5	3.5	3.0				2.8														
CYP1A1							7.8	2.6		4.5	2.3	2.2	1.6	−1.6						8.5	8.1			
CYP1A2		−1.7	1.6			−70.0	3.8	2.6		2.9	2.3	2.8								9.6	7.4			
CYP2B6	1.7		2.4			−22.2		2.2	−4.5				2.9											
CYP2C9																							−4.9	
CYP2C19				−6.3	−2.1	−28.6		2.0	−4.0				3.2	3.0					−4.4	1.8				
CYP2D6		−1.9																						
CYP2E1	−1.7														−6.2									
CYP3A4			8.4	−11.1						1.7		10.5	3.0	1.9		−8.7			−5.5					4.7
FMO3	−1.4	−1.8																	−6.3	−1.6				
FMO4				−4.0	−3.2	−6.4																		
FMO5				−7.2																				
**Oxidative Stress**																								
AASS										−2.6								−2.3	−17.4					
BDH2																			−5.4		−2.6			
GSTA3	−3.1			−34.4						2.0	−2.3		3.4			−4.4		−2.0						
GSTM4				−3.5	−3.1	−7.5											1.9		−6.5					
GPX1																			−3.4					
GPX2	1.5				−15.9					2.9	1.8		2.6	1.5					−1.6		−2.0		−1.8	−3.4
GPX3		−1.6								2.1		3.1	2.2					−2.3						
GPX5			2.3															2.7						
GPX6			2.3																					
CAT																	1.6		−8.0					
EPHX1	1.6			−2.2	−2.5	−8.9				2.5			1.8	1.6										
DUOX1		−3.1								2.3	1.8											2.5		
DUOX2		−2.3											4.1	−2.1								4.3		2.9
NQO1								1.9	2.9	2.6														
NUDT15											−2.1	2.0											−1.8	
PPP1R15B			7.3	2.0	3.9	26.4			8.2		−2.0	9.3						7.5			8.2			8.6
PRDX1									3.7			3.2						3.1						
TXNIP		−1.6												−1.9								2.8		
CTSB			3.6																				−4.4	
**Mitochondria Energy**																								
ACO1								−1.7			−2.6			−3.7			−2.5			−2.1			−2.9	
ACO2		−1.5																						
ACLY																						2.3		
ADK																			−5.8					
CYC1								1.4			1.3													
COX8A																			−6.1					
DLD															−27.3									
IDH1								−1.7																
IDH2		−1.7												−1.7										
IDH3B		1.3															−1.8							
MDH1	−1.5		3.1						3.1		−1.9	3.6			−2.9	−4.2		3.6					−1.9	
SDHC															−5.7					−1.7				
SUCLA2															−10.3									
SUCLG1 −1.4			3.3													−4.1								
UCP2				−2.4	−2.9	−5.1																		
**Heat Shock, ER Stress**																								
HSPA2																		−2.9				2.4		
HSPA4			1.8															1.9						
HSPA8														−1.9										
HSPA9	1.3			2.1	3.6	2.1																		
HSPA1A																				−1.9				
HSPA1B																		−2.2		−1.8				
HSP90AA1 1.5																				−1.5				
HSP90B1			4.3	2.5	2.0	10.9		−1.9							−25.2		−2.1	3.9		−1.6		1.9		6.4
HSPB1																							−2.6	
HSPB6																							2.3	
HSPB8																3.3		−2.9				2.6		
HSPD1										1.8			1.9	−2.1										
HSPE1																			−1.8	−1.6			−3.3	
HSF2						8.0																	−8.8	
HSPH1															−7.7								−1.8	
HERPUD1						7.0		−1.8									−2.0							
SEC62		−2.1	6.4						7.2		−3.4	7.9		−4.6				5.7					−2.7	9.6
SEL1																						2.0		
SEL1L			4.4	2.5	2.6	12.8			5.1			4.2						−1.6						6.9
SELS																						1.7	1.8	
SERP1		−2.7		2.4	3.2	7.1			3.4		−3.2			−2.7		−2.3	−4.3				3.6			6.2
ATF4				3.4	5.1	3.3				2.1													1.5	
ATF6				2.3	2.6	2.8																		
CRYAA																			−8.5					
CRYAB										1.9		2.8	−2.9					3.2	−3.4	−1.8			−3.8	
DDIT3		−1.9		4.9	16.3	24.8											−1.8							
DNAJB1																			−3.9	−1.5				
DNAJB6	1.4		2.3								1.4													
DNAJC3																	−1.7					2.4		
CNAJC5																						2.0		
DNAJC6			2.3	2.9	8.6	5.5													−3.1	−2.3				
DNAJA1																				−1.4				
DNAJA2	1.4							1.4																
DNAJA3										2.1			2.0											
DERL1				3.5	3.8	2.8																		
EDEM1																						2.5		
EIF2AK3				3.4	5.8	11.5																2.9		
ERO1LB						5.9		−1.6														1.9		
MBTPS2			2.4								−2.4	2.4		−1.5									−2.5	
NUCB1																							−2.1	
OS9																						2.4		
SYVN1				2.5	2.3	2.2																		
TCP1	1.2																							
UBE2G2	1.6						2.5																	
UBQLN2																							−2.8	
XBP1		−1.6																						
**Fatty Acid Metabolism**																								
ACAA1													−2.2			4.7	1.8		−34.0		−2.8			
ACAA2																2.4			−5.9		−2.5			
ACADM		−1.9	3.2							1.9	−2.1	3.4						4.1	−5.2					2.5
ACADSB				−7.3				1.7											−6.1					
ACADL															2.3				−7.2					
ACADVL										2.7	1.7				3.3	1.8						1.9		
ACAT1																			−6.2					
ACAT2				−1.9	−2.1	−3.5													−4.3		−2.2			
ACOT1			2.3																					
ACOT6																			−6.9					
ACOT7																			−17.9					
ACOT8	1.6																							
ACOT9																			−7.8					
ACOT12	−1.4			−3.9										1.6					−11.2					
ACOX1																	1.8		−8.4					
ACOX2				−5.9	−4.4	−12.5								1.5					−8.2					
ACOX3								1.6																
CPT1A	2.1									8.1	1.5		2.1			16.1	2.4	2.3				2.5		
APOA5																			−8.1					
APOE	−1.8	−1.7	3.3																					3.4
APOF		−2.2		−5.5	−5.2	−8.7													−4.9	−1.7				
DECR1																			−5.8					
ECHS1																			−4.9		−2.4			
EHHADH															−14.7		1.6		−5.4		−1.8			
HADHA										2.1						3.3	1.5		−3.1		−1.5			
HADHB										3.2									−2.3		−2.3			
ALB													−1.5	1.4	−3.8				−7.6					
**Steatosis, Cholestasis, Phospholipidosis**																								
ABCB1	1.5			2.8	2.8	4.1				2.2				1.5					−21.1					
ABCB4	−1.5	−1.5	−2.2					−1.6											−25.8		−2.0			
ABCC1																2.7								
ABCC2	1.7		2.4					2.6		3.2			2.5	1.6			1.9							
ABCC3	1.7						3.7	1.6		3.3			2.0			3.7								
OSTβ		−2.7		5.2													−2.4							
AQP																			−10.9					
AQP4																				−2.6				
ACACA										2.6			2.1									1.9		
SLCO1A2				−28.9	−7.9	−20.9		2.1						1.8										
SLC2A3																						2.5		
SLC10A1				−18.3							−2.6													
ADH1C				−11.3	−9.1	−7.4													−7.0					3.3
ALDH1A1			1.9	−2.4	−3.5	−10.7		2.2					2.5	1.6					−4.6					
ALDH2																			−5.7					
ASAH1																			−5.7					
ASNS				6.6	7.4	4.4				2.9														
CYP7A1			3.0	−51.1				4.7		−4.5						−3.3	2.3		−19.4	4.5			3.9	
CYP7B1		−2.8	6.0						4.8			6.4						4.6			4.9		−14.3	6.6
CD36																2.4			−5.2					
COMT																			−5.2					
DNM1								2.4																
ENO1																						1.8		
FABP1	−2.3	−1.6	−2.3			−22.0		−1.7	−3.5	7.2					−3.2	5.6				−1.5	−4.3			−3.4
FASN		−2.2																						
FXC1				2.5	4.0	2.5																		
GPD1		−1.7						2.1									1.9		−1.8	1.9				
HAAO				−14.1																				
HLA−DRB1																					−2.6			
ICAM1															−15.8			−2.1						
INHBE																						2.7		
KHK				−4.2																	−2.4			
LPL			1.9	3.1	2.9	4.4										4.5								
MTTP				−2.8	−5.7	−11.5																		
NR0B2																					−1.5			
NR1H4																					−2.1			
PCCA	3.6									5.1			3.7			8.5						5.2		
PNPLA3																							−2.6	
RDX															−12.6									
SCD		−2.9								3.9											−4.1			
SREBF1		−3.4																					−3.7	
SC4MOL	3.3	−1.6								6.8	−1.9		6.4			6.0						6.6	−2.3	
S100A8															−6.5									
TAGLN																						2.0		
TGFB1																					−4.8		−1.9	
UGT1A1										3.3			1.9									1.8	−4.3	
UGT2A1			2.3																					
UGT2B4 2.1			11.4					2.2	4.5	2.7	−3.9	6.0						4.1			5.8		−3.2	4.9
WIPl1				2.9	4.0	6.7																1.8		
**DNA damage, repair**																								
AHR																			−5.2					
APEX1																			−5.1					
BRCA1																						3.2		
BRCA2								1.8	3.2			2.8		−2.0										
CDKN1				2.1	3.8	3.3													−4.6		−2.4			
CDKN1A																								−3.2
CHEK1								1.8										−1.9						
CHEK2															−8.4				−4.2		−2.3			−2.3
DDIT3																				−1.6				
ERCC1			4.6	2.5	3.8	24.6			6.7			6.7						2.5			7.8		−2.1	8.4
ERCC6											−1.8													
HPRT1								1.4																
MGMT								1.5																
MLH1																						2.1		
MSH2									3.9		−2.6	3.5						3.4		−1.7	3.2		−4.4	3.6
OGG1								1.6																
POU3F3			2.3																					
PRKDC											−2.9						−2.2							
RAD51																							−2.9	
XPA																						−3.3		
**Apoptosis**																								
AKT1																						2.1		
APAF1																						2.2		
BAK1																			−6.0					
BID			2.8											1.4				2.3						
BIRC3								−1.7									−2.0							
BCL2L1																						2.2	−1.9	
BCL2L11		−1.9																						
CASP1																			−4.7	−1.6				
CASP3															−3.9									
CASP8										2.4	1.6													
FADD			2.5	2.1	3.3	6.8			2.7	1.8		2.9						2.2			2.7	2.2		2.9
FAS			5.4			11.0			8.3		−2.5	8.0						5.5	−3.1		6.9			7.6
FASLG	−2.1			−8.3							2.1													
GADD45A				2.5	11.5	30.7							−3.5		−3.9				−8.0					
MK167			−4.7			−30.7		−2.5							−4.3			−3.1		−3.7	−5.9		−4.6	−27.7
TNFSF10				−5.0	−12.0	−24.8												−2.6						
TNFRSF10A				3.8	2.7	6.3							2.7									2.2		2.5
TNFRSF10B				2.9	3.6	4.1																		
XIAP				2.0	2.4	5.5									−7.9			3.3			2.8			3.3
**Necrosis**																								
ATP6V1G2		−3.2																−2.5						
BMF																2.8	2.2					2.2		
CYLD						4.2																		
DEFB1																	−2.6							
EIF5B										1.9									−4.3					
FOXl1			2.3																					
GALNT5			2.3																					
HOXA3			2.2																					
JPH3			2.3																					
KCNIP1			2.3																					
NUDT13		−3.4	3.9						2.6			3.0						2.8			2.9			4.8
PVR																						2.4		
RAB25																	−2.9			−2.2				
TMEM57															−16.2								1.8	
TNFAIP8L1										2.6													−2.0	
S100A7A			2.3																					
**Immune Response, Inflammation**																								
AHSG				−2.0	−3.4	−4.2		−1.7	−2.4															
IL1A			2.2																					
IL1B								−2.9									−10.2	−2.5		−3.5	−2.4		−8.2	−3.9
IL2			2.4																					
IL4															56									
IL6		−2.5													−4.2									−4.9
IL10																						2.8		
C3		−1.9													−33.3		−1.8							
C9				−6.3	−3.8	−7.6																2.0		
CD4																						2.8	−2.8	
CD80										2.2	1.6			1.8	−5.7									
CD86												2.5							−4.5					
CTSE																2.1	−2.9							
EP300												2.2						2.5			2.2			2.5
HRG			4.1																				−3.3	
HRT2A				2.1	2.1	2.3																		
IFNG														2.6						−2.4		−3.3		
KLF1																		−2.2						
LY6D			2.6																					
LYZ								−2.2							−4.9		−2.6			−1.9			−1.9	
PON1	−1.7			−7.0										1.8							−2.5			
PTGS2					16.9	34.8		−2.1																
SOD1														1.5										
TRIM10									4.0			4.1						3.9			3.3			3.6

**Table 5:** Summary of oxidative stress and mitochondria function markers, ATP and GSH levels (↑ = increase, ↓ = decrease) plus gene expression pathways affected by each drug. Gene categories listed in each column represent the percent of total gene expression changes by each drug.

Drug	Dose	Human Liver Slice ATP Levels 72 h	Human Liver Slice GSH Levels 72 h	Oxidative Stress, Mitochondria Energy, Heat Shock/ER Stress, 48 h	DNA Damage/Repair, Apoptosis, Necrosis, Inflammation & Immune, 48 h	FA Metabolism, Steatosis, Cholestasis, Inflammation Immune, 48 h
APAP	1 mM	↓ 16%–25%, *n* = 3	↑ 21%–39%, *n* = 3	34%	23%	40%
DCF	1 mM	↓ 50%–85%, *n* = 3	↓ 30%–50%, *n* = 2	34%	27%	38%
ETM	100 μM	↓ 20%, *n* = 2	↑ 13%–24%, *n* = 2	35%	31%	43%
DTL	10 μM	↓ 19%, *n* = 3	↑ 31%–68%, *n* = 2	23%	25%	51%
CSA	10 μM	↓ 17%–30%, *n* = 2	↓ 30%, *n* = 1	37%	38%	35%
MMI	500 μM	↓ 14%–33%, *n* = 2	↑ 30%–49%, *n* = 3	28%	32%	37%
TBF	100 μM	↓ 25%, *n* = 1; ↑ 30%, *n* = 1	↑ 20%–39%, *n* = 2	36%	25%	35%
CBZ	100 μM	↓ 17%, *n* = 1	↑ 20%–30%, *n* = 2	33%	23%	44%
